# Loss of function of BRCA1 promotes EMT in mammary tumors through activation of TGFβR2 signaling pathway

**DOI:** 10.1038/s41419-022-04646-7

**Published:** 2022-03-02

**Authors:** Feng Bai, Chuying Wang, Xiong Liu, Daniel Hollern, Shiqin Liu, Cheng Fan, Chang Liu, Sijia Ren, Jason I. Herschkowitz, Wei-Guo Zhu, Xin-Hai Pei

**Affiliations:** 1 Guangdong Provincial Key Laboratory of Regional Immunity and Diseases, International Cancer Center, Marshall Laboratory of Biomedical Engineering, Shenzhen University Health Science Center, Shenzhen, 518060 China; 2 Department of Pathology, Shenzhen University Health Science Center, Shenzhen, 518060 China; 3 Dewitt Daughtry Family Department of Surgery, University of Miami, Miami, FL 33136 USA; 4 The Second Affiliated Hospital of Xi’an Jiaotong University, Xi’an, Shaanxi 710061 China; 5 Department of Anatomy and Histology, Shenzhen University Health Science Center, Shenzhen, 518060 China; 6 Lineberger Comprehensive Cancer Center, University of North Carolina at Chapel Hill, Chapel Hill, NC 27599 USA; 7 Peking University First Hospital, Beijing, 100034 China; 8 Department of Biochemistry and Molecular Biology, International Cancer Center, Shenzhen University Health Science Center, Shenzhen, 518060 China; 9 Present Address: Department of Biomedical Sciences, University at Albany, Rensselaer, NY 12144 USA

**Keywords:** Breast cancer, Oncogenesis

## Abstract

BRCA1 deficient breast cancers are aggressive and chemoresistant due, in part, to their enrichment of cancer stem cells that can be generated from carcinoma cells by an epithelial-mesenchymal transition (EMT). We previously discovered that BRCA1 deficiency activates EMT in mammary tumorigenesis. How BRCA1 controls EMT and how to effectively target BRCA1-deficient cancers remain elusive. We analyzed murine and human tumors and identified a role for Tgfβr2 in governing the molecular aspects of EMT that occur with Brca1 loss. We utilized CRISPR to delete Tgfβr2 and specific inhibitors to block Tgfβr2 activity and followed up with the molecular analysis of assays for tumor growth and metastasis. We discovered that heterozygous germline deletion, or epithelia-specific deletion of Brca1 in mice, activates Tgfβr2 signaling pathways in mammary tumors. BRCA1 depletion promotes TGFβ-mediated EMT activation in cancer cells. BRCA1 binds to the TGFβR2 locus to repress its transcription. Targeted deletion or pharmaceutical inhibition of Tgfβr2 in Brca1-deficient tumor cells reduces EMT and suppresses tumorigenesis and metastasis. BRCA1 and TGFβR2 expression levels are inversely related in human breast cancers. This study reveals for the first time that a targetable TGFβR signaling pathway is directly activated by BRCA1-deficiency in the induction of EMT in breast cancer progression.

## Introduction

Clinically, breast cancer comprises three main subtypes: human epidermal growth factor receptor 2 (HER2) positive breast cancer, hormone receptor [estrogen receptor (ER) and/or progesterone receptor (PgR)]-positive breast cancer, and triple-negative breast cancer (TNBC), the latter of which lacks expression of ER, PgR, and HER2 [1, 2]. Gene-expression analyses have categorized human breast tumor into six intrinsic subtypes: basal-like (BL), claudin-low (CL), Her2-enriched, luminal A, luminal B, and normal breast-like, each of which has unique biological and prognostic features [3]. Of these subtypes of breast cancer, the CL subtype is a TNBC characterized by the high enrichment for epithelial to mesenchymal transition (EMT) markers and cancer stem cell (CSC)-like features [3, 4]. Basal-like breast cancer (BLBC) accounts for approximately 70% of TNBCs and is a leading cause of cancer deaths worldwide. The high mortality rate of BLBCs can be attributed to the aggressive and metastatic capacity of these tumors and the limited number of effective therapeutic options. BLBCs are highly aggressive and metastatic in part due to their enrichment of CSCs, which are thought to drive clinical relapse and metastasis [5, 6]. CSCs are more resistant to radio- and chemotherapy and can be generated from carcinoma cells by an EMT program [7–9], which is a process whereby epithelial cells lose many of their epithelial characteristics and acquire mesenchymal features [9]. While some molecular regulators of EMT have been identified [10], therapeutically targetable mechanisms controlling EMT in BLBCs remain elusive.

TGFβ signaling plays a key role in inducing and maintaining the mesenchymal and stem cell states of multiple tissues, including breast tissues [11]. TGFβ ligands bind to their receptors, TGFβR1/2, which then activate downstream effectors through SMAD-dependent and SMAD-independent pathways, to activate EMT and drive CSC function [12, 13]. Further, the expression of TGFβR2 is enhanced in ER-negative tumor cells, and is reduced in ER-positive tumor cells [14, 15]. Overexpression of TGFβR2 or upregulation of TGFβ signature genes is associated with lung metastasis and lower survival rates in BLBCs [16, 17]. Together, these prior studies demonstrate an association between TGFβR2 and aggressive tumor phenotypes. Yet, the role of TGFβR2 and its status as a putative therapy target in ER-negative BRCA1 deficient tumors is currently unknown.

We and others have reported that BRCA1 deficiency activates p16, p18, and RB, key proteins in controlling cell cycle progression [18–21]. Loss of *Brca1* in *p16* or *p18* deficient mice activates EMT and induces BLBCs, whereas, loss of p18 induces luminal type mammary tumors [18–20, 22]. In this study, we follow up on these results to identify the molecular mechanisms by which Brca1 loss promotes EMT.

## Results

### Germline deletion of Brca1 activates Tgfβ signaling and EMT with enhanced expression of Tgfβr2 in mammary tumor cells

**Fig. 1:**
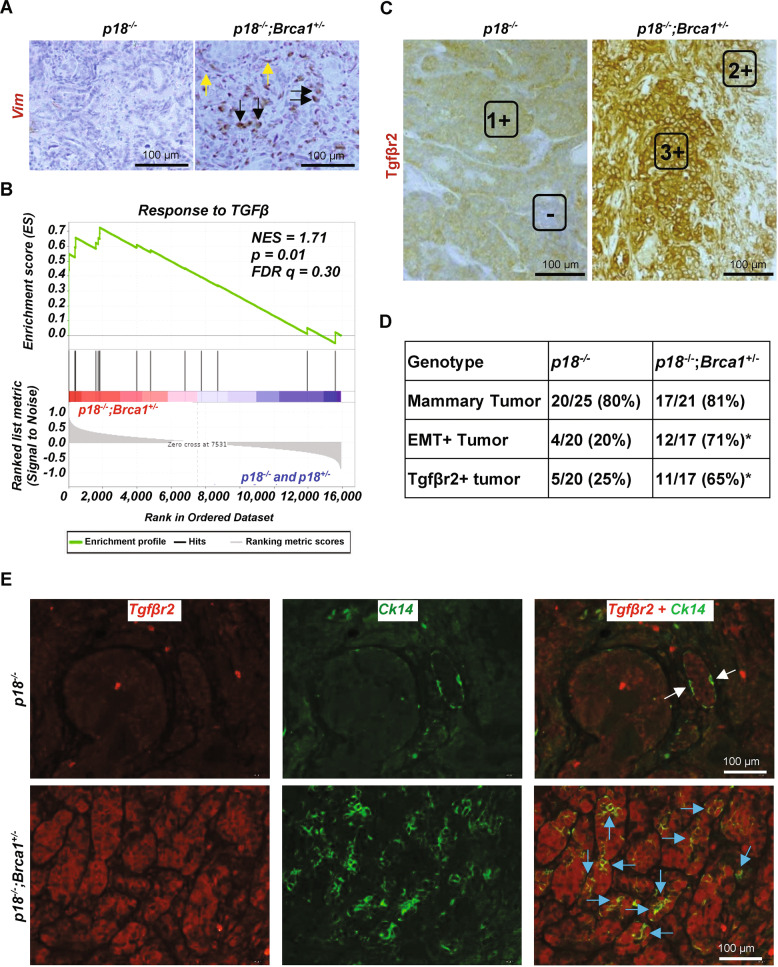
Heterozygous germline deletion of Brca1 in p18 deficient mice activates EMT and Tgfβ signaling with an increase of Tgfβr2 expression in mammary tumor cells. **A** Representative IHC analysis of mammary tumors with antibodies against Vim. Vim positive tumor cells (Black arrows) and stromal cells (Yellow arrows) are indicated. **B** Microarray analysis of mammary tumors. GSEA enrichment plot for a signature for Tgfβ signaling activity. NES: Normalized Enrichment Score (NES), Nominal *p* value (p), and False Discovery Rate q-value (FDR q) were detected comparing *p18*^−/−^;*Brca1*^+/−^ tumors (*n* = 10) to *p18*^−/−^ and *p18*^+/−^ tumors including nine *p18*^−/−^ and one *p18*^+/−^ tumors. **C** Representative IHC analysis of mammary tumors from a *p18*^−/−^ and *p18*^−/−^; *Brca1*^+/−^ mice. To quantify the Tgfβr2 positive tumor cells, the intensity of Tgfβr2 antibody-specific staining by IHC in tumor cells were categorized into -, 1 + , 2 + , and 3 + . The representative images in the boxed area for each category were shown. **D** Summary of mammary tumors in mice with Balb/c background. EMT + tumors are tumors that are positive for at least two EMT markers (decreased E-Cad, increased Vim, Fn1, or CD29), and two EMT-TFs (Twist, Snail, Slug, Foxc2 or p-Fra1) in >2% tumor cells. Tgfβr2 + tumors are tumors that are positive for Tgfβr2 with 2+ or 3+ intensity in >20% tumor cells. The asterisk (*) denotes a significance from *p18*^*−/−*^*; Brca1*^*+/−*^ and *p18*^*−/−*^ tumors by two-tailed Fisher’s exact test. **E** Representative immunofluorescent staining of mammary tumors from a *p18*^−/−^ and *p18*^−/−^; *Brca1*^+/−^ mice. Note that the majority of Tgfβr2 positive *p18*^−/−^; *Brca1*^+/−^ tumor cells were co-stained with Ck14 (blue arrows). Ck14 singly positive basal epithelial cells (white arrows) in the normal gland are indicated.

We previously demonstrated that heterozygous germline deletion of Brca1 in p18 null mice activated EMT and led to BLBCs with enhanced CSC populations along with the increased potential for metastasis {Figs. 1A, S1A–C, and details in [18, 19, 22]}. To identify the molecular determinants for activation of EMT in Brca1 deficient mammary tumors in an unbiased manner, we performed microarray analysis. GSEA revealed enrichment of a signature for active Tgfβ signaling in *p18*^−/−^; *Brca1*^+/−^ tumors when compared to *p18*^−/−^ and *p18*^+/−^ tumors (Fig. 1B). Consistent with activation of known downstream pathways by BRCA1 loss [23, 24], signatures for NF-КB pathway activity were also enriched in *p18*^−/−^; *Brca1*^+/−^ tumors (Fig. S1D). Most (9/10) *p18*^−/−^; *Brca1*^+/−^ tumors expressed high levels of Tgfβr2, whereas most (8/10) p18^−/−^ and p18^+/−^ tumors expressed a low level of Tgfβr2. Analysis of tumors after removal of an outlier p18^−/−^;Brca1^+/−^ tumor, a p18^+/−^ tumor and an outlier p18^−/−^ sample, revealed that Tgfβr2 mRNA levels in p18^−/−^;Brca1^+/−^ tumors were significantly higher than those in p18^−/−^ tumors (Fig. S2). Interestingly, we found that Tgfβr2 mRNA did not elevate in K14-Cre;p53^f/f^;Brca1^f/f^ mammary tumors, suggesting that upregulation of Tgfβr2 may be unique to Brca1 germline mutant tumors also deficient in p18. We also noted that within individual intrinsic subtypes in genetically engineered mouse models existed a range of expression values for Tgfβr2, with statistically significant association of elevated mRNA in CL subtype (Fig. S2).

We performed IHC and found a significantly enhanced frequency of Tgfβr2-positive tumors when *p18*^−/−^; *Brca1*^+/−^ tumors were compared with *p18*^−/−^ tumors. Furthermore, most (11/12) *p18*^−/−^; *Brca1*^+/−^ tumors with EMT features expressed high levels of Tgfβr2 (Fig. 1C, D). Interestingly, we observed an insignificantly increased frequency of *p18*^−/−^; *Brca1*^+/−^ tumors that were Tgfβr1-positive when compared with *p18*^−/−^ tumors (7/14 positive in *p18*^−/−^; *Brca1*^+/−^ tumors versus 6 /14 positive in *p18*^−/−^ tumors) (data not shown). Since TGFβR2 is also expressed in mammary stromal cells [12, 14], we determined the expression of Tgfβr2 and Ck14, an epithelial marker, in tumor cells. We noticed that the majority of Tgfβr2 positive *p18*^−/−^;*Brca1*^+/−^ tumor cells were co-stained with Ck14, indicative of the epithelium origin of the Tgfβr2 positive tumor cells (Fig. 1E). These results imply a possible negative correlation between Brca1 and Tgfβr2 in mouse mammary tumors.

We examined the expression of Tgfβr1/2 in mouse embryos in which Tgfβ signaling and EMT play a critical role in development [9]. The expression of Tgfβr2, along with a few EMT-inducing transcription factors (EMT-TFs), was significantly increased in *p18*^−/−^; *Brca1*^+/−^ embryos relative to their *p18*^−/−^ counterparts. We noted that the expression of Tgfβr1 was not different in this same comparison (Fig. S3). Together, our data depicts a clear relationship between Brca1, Tgfβr2, and EMT in our murine system.

### Specific deletion of Brca1 in mammary epithelia enhances Tgfβr2 expression and activates EMT in mammary tumors

We generated *p18*^*−/−*^*; Brca1*^*f/f*^; MMTV-Cre (*p18*^*−/−*^*; Brca1*^*MGKO*^) mice, in which Brca1 was specifically deleted in mammary epithelial cells (MECs). 47% (7/15) of *p18*^−/−^ and 73% (11/15) of *p18*^*−/−*^*; Brca1*^*MGKO*^ mice developed mammary tumors [19]. Interestingly, we observed lung metastasis in *36%* (*4/11)* of *p18*^*−/−*^*; Brca1*^*MGKO*^ tumor-bearing mice, but did not observe metastasis in the lungs of mice with *p18*^−/−^ tumors. Consistent with prior reports of a linkage between EMT and metastatic potential [25], significantly more *p18*^*−/−*^*; Brca1*^*MGKO*^ tumors than *p18*^*−/−*^ tumors were positive for EMT markers including vimentin (Vim), fibronectin (Fn), Twist, and p-Fra1 {details in [19]}. These data demonstrate that specific deletion of Brca1 in p18 null mammary epithelia induces a malignant mammary tumor phenotype with EMT features.

**Fig. 2:**
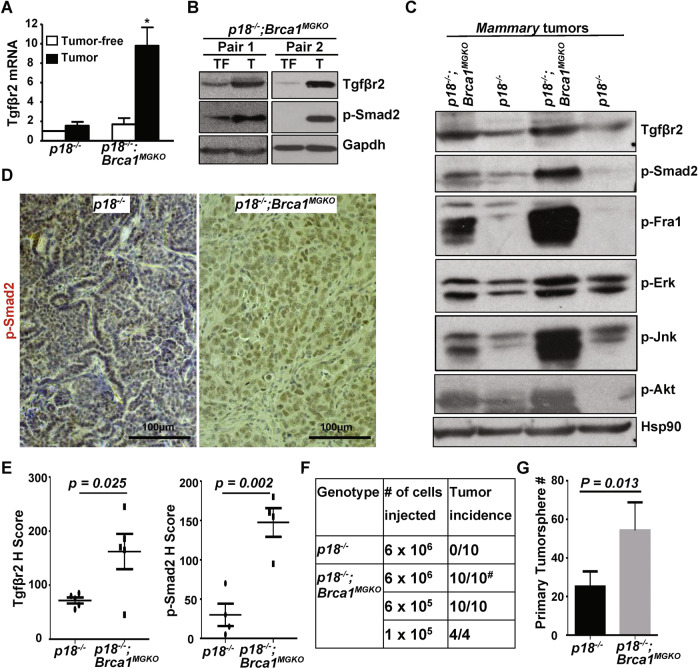
Deletion of Brca1 in p18^−/−^ epithelia activates Tgfβr2 signaling pathways and promotes tumor-initiating potential. **A, B** Tumors (T) from *p18*^−/−^ and *p18*^−/−^; *Brca1*^MGKO^ mice were analyzed by qRT-PCR (A) and western blot (**B**). Tumor-free mammary glands (TF) from the same mouse were used as controls. Data in (**A**) represent the mean ± SD of three tumors in each group. The asterisk (*) denotes a significance from tumors and tumor-free tissues of the same genotype by a two-tailed, unpaired *T* test. **C** Four representative tumors from *p18*^−/−^ and *p18*^−/−^; *Brca1*^MGKO^ mice were analyzed by western blot. **D** Representative immunostaining of mammary tumors from a *p18*^−/−^ and *p18*^−/−^; *Brca1*^MGKO^ mice. **E** *p18*^−/−^ and *p18*^−/−^; *Brca1*^MGKO^ mammary tumors were analyzed by IHC, and H-scores for Tgfβr2 and p-Smad2 were calculated. The results represent the mean ± SD of five individual tumors per group for Tgfβr2 and four individual tumors per group for p-Smad2. **F** *p18*^−/−^ and *p18*^−/−^; *Brca1*^MGKO^ mammary tumor cells were transplanted into MFPs of NSG mice. Four weeks later, recipient mice with tumors generated (>0.5 cm^3^ in size) were analyzed. ^#^five tumor-bearing mice displayed lung metastasis by H.E. analysis. **G** 3 ×10^4^ *p18*^−/−^ and *p18*^−/−^; *Brca1*^MGKO^ mammary tumor cells were cultured to generate primary tumorspheres in 10 days. The number of spheres large than 50 μm was quantified from triplicate experiments. The results represent the mean ± SD of three individual tumors per group. Statistical significance in (**E**) and (**G**) was determined by a two-tailed, unpaired *T* test.

We compared *p18*^*−/−*^*; Brca1*^*MGKO*^ tumors to tumor-free mammary tissues, and to *p18*^*−/−*^ tumors. Both Tgfβr2 mRNA and protein levels, as well as the level of phosphorylated-Smad2 (p-Smad2), were significantly increased in *p18*^*−/−*^*; Brca1*^*MGKO*^ tumors relative to those in tumor-free mammary tissues of the same mice. In comparison, Tgfβr2 mRNA was moderately enhanced in *p18*^*−/−*^ tumors relative to that in tumor-free mammary tissues of the same mice (Fig. 2A, B). Furthermore, when compared with *p18*^*−/−*^ tumors, most *p18*^*−/−*^*; Brca1*^*MGKO*^ tumors expressed an increased level of Tgfβr2 and p-Smad2, as well as p-Akt, p-Erk, and p-Jnk, all of which are Smad-independent downstream effectors activated by Tgfβ-Tgfβr2 signaling (Fig. 2C). Immunostaining revealed that Tgfβr2 and p-Smad2 were highly expressed in *p18*^*−/−*^*; Brca1*^*MGKO*^ tumor cells, and that the expression pattern of Tgfβr2 in *p18*^*−/−*^*; Brca1*^*MGKO*^ tumors was similar with the pattern in *p18*^*−/−*^*; Brca1*^*+/−*^ tumors (Figs. 1C, E, 2D). When the positive cells were quantified, we found that H scores for both Tgfβr2 and p-Smad2 in *p18*^*−/−*^*; Brca1*^*MGKO*^ tumor cells were significantly more than the H scores in *p18*^*−/−*^ counterparts (Fig. 2E). These data confirm that during mammary tumorigenesis, loss of Brca1 activates Tgfβr2 signaling pathways in an epithelium-autonomous manner.

### BRCA1 depletion enhances tumor initiation potential and promotes TGFβ-mediated EMT activation in breast cancer cells

We previously reported that CSC-enriched cells were expanded in p18^−/−^; Brca1^MGKO^ mammary tumors relative to their p18^−/−^ counterparts [19], and that p18^−/−^; Brca1^MGKO^ tumor cells displayed features that had undergone EMT [26]. We tested the tumor-initiating capacity of tumor cells and found that as low as 1 ×10^5^ of p18^−/−^; Brca1^MGKO^ cells were able to regenerate tumors, whereas as high as 6 × 10^6^ of p18^−/−^ cells did not yield tumors. Notably, half of the mammary tumors generated by 6 × 10^6^ p18^−/−^; Brca1^MGKO^ tumor cells metastasized to the lung (Fig. 2F). Furthermore, we detected significantly more primary tumorspheres formed by p18^−/−^; Brca1^MGKO^ tumor cells than those done by p18^−/−^ tumor cells (Fig. 2G). Together, these illustrate that loss of Brca1 in breast cancer cells enhances the CSC population and its property in tumor initiation.

**Fig. 3:**
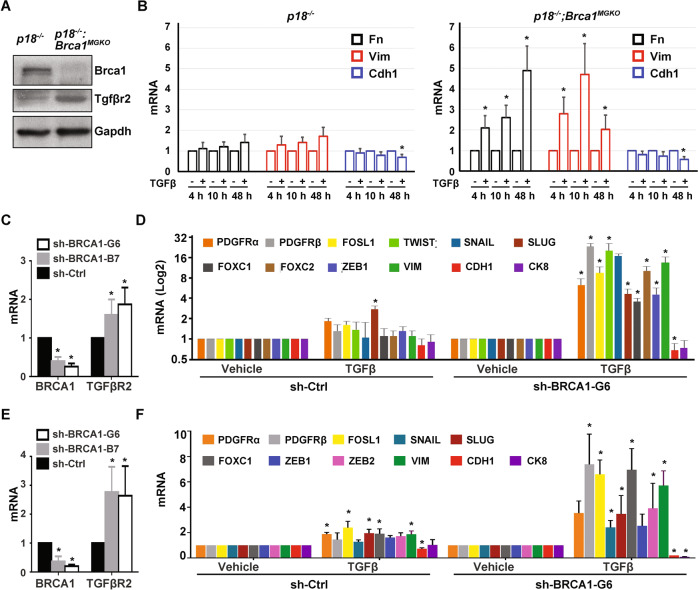
BRCA1 depletion sensitizes tumor cells to TGFβ-mediated EMT activation. **A**, **B** Representative *p18*^−/−^ and *p18*^−/−^; *Brca1*^MGKO^ mammary tumor cells were analyzed by western blot (**A**) or treated with vehicle or TGFβ for different time periods, and then analyzed by qRT-PCR (**B**). Data in (**B**) represent the mean ± SD from duplicates of two independent experiments from two different pairs of primary tumor cell lines. **C**, **E** MCF7 (**C**) and MDA-MB-231 (**E**) cells were infected with either pGIPZ-empty (sh-Ctrl.) or pGIPZ-shBRCA1 targeting different sequences of human *BRCA1* (sh-BRCA1-B7 and sh-BRCA1-G6). Cells stably expressing sh-Ctrl or shBRCA1 were analyzed by qRT-PCR. Data represent the mean ± SD from triplicates of each of the two independent experiments. **D**, **F** mRNA levels in MCF7-sh-Ctrl and MCF7-sh-BRCA1-G6 (**D**), or MDA-MB-231-sh-Ctrl and MDA-MB-231-sh-BRCA1-G6 (**F**) cells treated with TGFβ for 10 (**D**) or 24 (**F**) hours were analyzed. Data represent the mean ± SD from duplicates of two independent experiments. The asterisk (*) in (**B**) (**D**) and (**F**) denotes a statistical significance from vehicle- and TGFβ-treated samples determined by a two-tailed, paired *T* test. The asterisk (*) in (**C**) and (**E**) denotes a statistical significance from sh-BRCA1 and sh-Ctrl samples determined by a two-tailed, paired *T* test.

To test if Tgfβr2 activation promotes EMT in Brca1 deficient tumor cells, we used a TGFβ challenge assay. We confirmed the increase of Tgfβr2 expression in p18^−/−^; Brca1^MGKO^ tumor cells relative to that in p18^−/−^ cells (Fig. 3A). In p18^−/−^ cells, TGFβ treatment resulted in modest changes in EMT marker genes (increase of mesenchymal and decrease of epithelial genes), which was likely due to the diminished Tgfβr2 expression observed in these tumor cells. However, in p18^−/−^; Brca1^MGKO^ cells, the dramatic elevation of EMT markers was noted (Figs. 3B, S4A). We then knocked down BRCA1 in MCF7, T47D, and MDA-MB-231 cell lines, all of which are BRCA1 wild type (Wt) [19]. We discovered that the depletion of BRCA1 stimulated the expression of TGFβR2 mRNA. In response to TGFβ treatment, BRCA1-depleted cells, but not control cells, expressed a distinctly lower level of epithelial markers (CDH1, CK8) and higher level of mesenchymal markers (PDGFRβ, VIM), as well as EMT-TFs including FOSL1 (encoding FRA1), TWIST, and SNAIL (Figs. 3C–F, S4B, C). These results indicate that BRCA1 deficiency in cancer cells stimulates TGFβR2 expression and promotes TGFβ-mediated EMT activation.

### BRCA1 suppresses transcription of TGFβR2

**Fig. 4:**
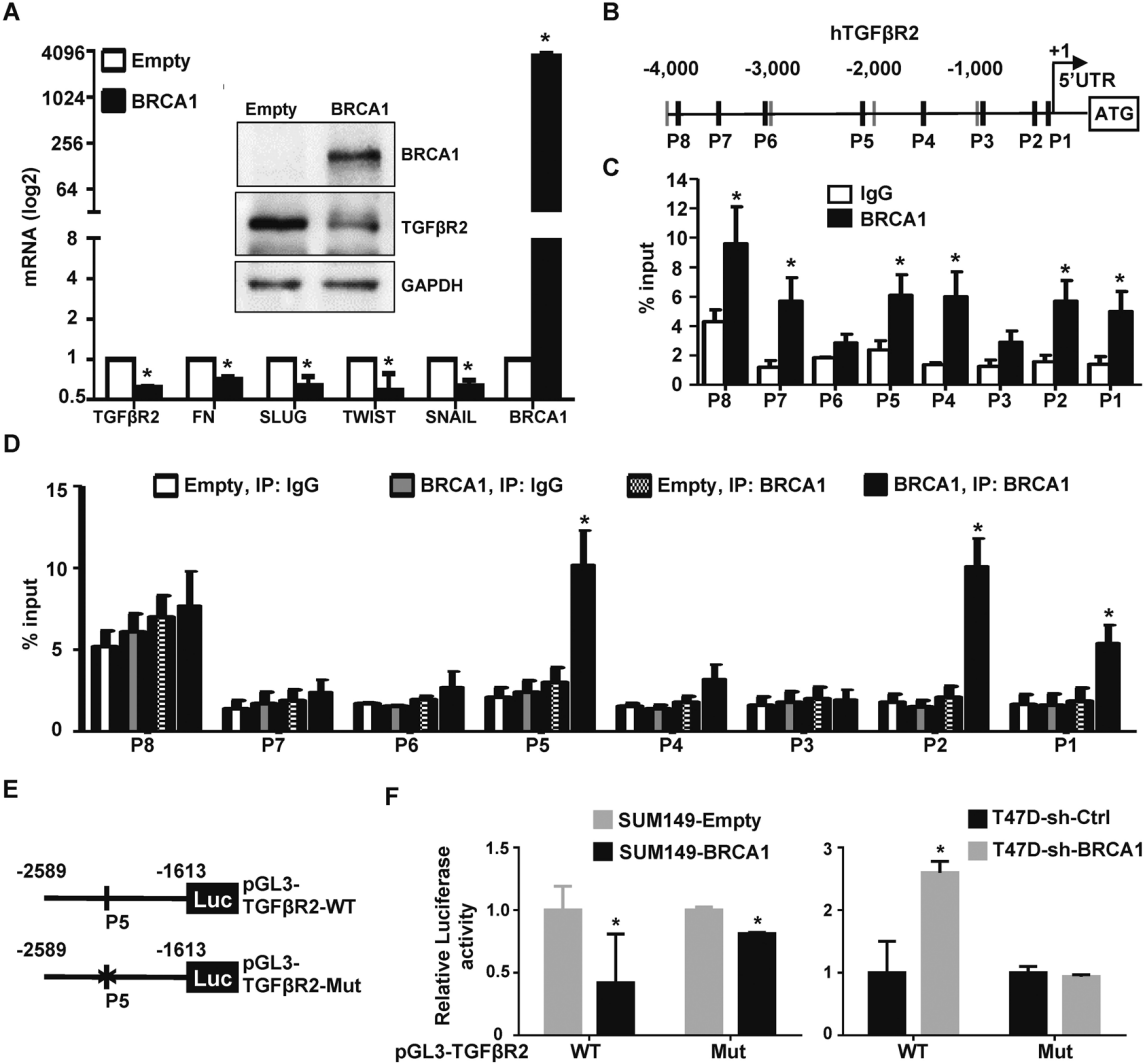
BRCA1 binds to TGFβR2 locus and represses expression of TGFβR2 and EMT-associated genes. **A** SUM149 cells were transfected with pBabe-empty (Empty) or pBabe-HA-BRCA1 (BRCA1). The expression of genes indicated was determined by western blot and/or qRT-PCR 72 h after transfection. Data represent the mean ± SD from triplicates of two independent experiments. The asterisk (*) denotes a statistical significance from empty- and BRCA1-expressing samples determined by a two-tailed, paired T test. **B** Diagram showing the location of putative BRCA1 binding sites (dark black bars) in the human TGFβR2 gene, and that of primers (P1 - P8) used for ChIP analysis. +1, transcription start site. **C** ChIP analysis of endogenous BRCA1 binding to putative BRCA1 sites on the TGFβR2 locus in T47D cells. Normal IgG was used as a negative control. The ratio of binding signal to input was compared. Data represent the mean ± SD from triplicates of two independent experiments. The asterisk (*) denotes a statistical significance from IgG and anti-BRCA1 immunoprecipitated samples by a two-tailed, unpaired T test. **D** ChIP analysis of exogenous BRCA1 binding to the TGFβR2 locus in HCC1937 cells transfected with pBabe-empty (Empty) or pBabe-HA-BRCA1 (BRCA1). Normal IgG was used as a negative control. The ratio of binding signal to input was compared. Data represent the mean ± SD from triplicates of two independent experiments. The asterisk (*) denotes a statistical significance from Empty- and BRCA1-expressing samples immunoprecipitated by anti-BRCA1 by a two-tailed, unpaired T test. **E** A schematic representation of WT and Mut TGFβR2 promoter-reporter constructs. Numbers represent a position relative to the transcription start site and a letter (X) denotes a mutated GATA3 binding site. The location of primers used for ChIP analysis is shown. **F** T47D-sh-Ctrl and T47D-sh-BRCA1 cells were transfected with Renilla and pGL3-TGFβR2-WT or pGL3-TGFβR2-Mut (right panel), and SUM149 cells were transfected with Renilla and pGL3-TGFβR2-WT or pGL3-TGFβR2-Mut, as well as pBabe-empty or pBabe-HA-BRCA1 (left panel), which were then collected after 48 h and assayed for luciferase activity. Data represent the mean ± SD from triplicates of two independent experiments. The asterisk (*) denotes a statistical significance from BRCA1 and empty (left panel), or sh-BRCA1 and sh-Ctrl (right panel) samples determined by a two-tailed, paired *T* test. SUM149, BRCA1 mutant (Mut); T47D, BRCA1 wild type (Wt).

We transfected BRCA1 into BRCA1-mutant cell lines, HCC1937 and SUM149, respectively. The introduction of functional BRCA1 led to lower mRNA and protein levels of TGFβR2. Consistent with our previous finding [19], overexpression of BRCA1 resulted in a decrease of mesenchymal markers and EMT-TFs (Figs. 4A, S5). These data confirm that BRCA1 represses the expression of TGFβR2 and inhibits EMT.

We and others have shown that GATA3 recruits BRCA1 to its binding sites in the promoters of FOXC1/2 and TWIST genes to repress their transcription [19, 27]. Analysis of the TGFβR2 locus revealed at least twelve putative BRCA1/GATA3 binding sites (Fig. 4B). We performed ChIP assays using 8 pairs of primers that cover all twelve putative BRCA1/GATA3 binding sites. Six out of eight amplicons (P1, P2, P4, P5, P7, and P8) in T47D cells and three out of eight amplicons (P1, P2, and P5) in HCC1937 cells transfected with BRCA1 were specifically enriched in the immunoprecipitation of BRCA1 (Fig. 4C, D). Together with the experimental evidence that BRCA1 represses TGFβR2, we attribute these observations to direct repression by the binding of BRCA1 to the TGFβR2 locus.

We generated TGFβR2 promoter-luciferase fusion plasmids, pGL3-TGFβR2-WT containing a promoter region that covers P5 amplicon, and pGL3-TGFβR2-Mut in which a BRCA1/GATA3 binding site of pGL3-TGFβR2-WT was mutated (Fig. 4E). We transfected the plasmids into SUM149 cells and found that relative to an empty control; ectopic BRCA1 drastically reduced the activity of pGL3-TGFβR2-WT promoter by ~60%. On the other hand, the transfection slightly reduced the activity of pGL3-TGFβR2-Mut promoter by ~18% (Fig. 4F, left). Furthermore, we detected that the activity of pGL3-TGFβR2-WT promoter was significantly increased in T47D-sh-BRCA1 cells relative to their T47D-sh-Ctrl cell counterparts. However, this was not the case when we examined the activity of pGL3-TGFβR2-Mut promoter (Fig. 4F, right). These data reaffirm our conclusion that BRCA1 binds to BRCA1/GATA3 sites in TGFβR2 promoter to suppress its activity.

### Targeted deletion of Tgfβr2 in Brca1 deficient tumor cells suppresses EMT and inhibits tumor initiation and metastasis

**Fig. 5:**
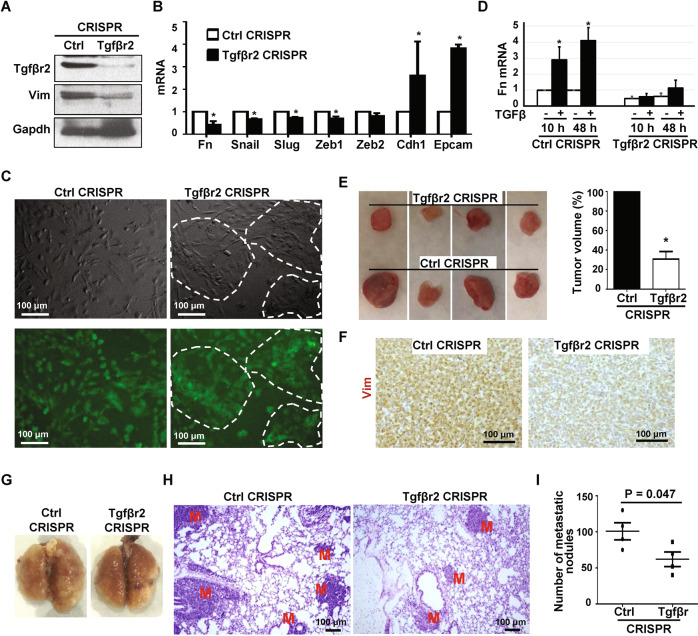
Deletion of Tgfβr2 in Brca1-deficient tumor cells inhibits EMT and suppresses tumorigenesis and metastasis. **A**–**C** *p18*^−/−^; *Brca1*^MGKO^ tumor cells were transfected with Tgfβr2 (Tgfβr2 CRISPR) and Control (Ctrl CRISPR) Double Nickase plasmids then selected with puromycin for 3 days. Tgfβr2- and control-knockout cells were then analyzed by western blot (**A**), Q-RT-PCR (B), and microscope for cell morphology (**C**). The areas with epithelial-like cells are indicated in (**C**). **D** Tgfβr2- and Ctrl-knockout *p18*^−/−^; *Brca1*^MGKO^ mammary tumor cells were treated with vehicle or TGFβ for different time periods, and the expression of Fn was then determined. Data in (**B**) and (**D**) represent the mean ± SD from duplicates of two independent experiments. The asterisk (*) in (**B**) denotes a statistical significance from Tgfβr2- and Ctrl-knockout samples, and in (**D**) denotes a statistical significance from TGFβ treated and vehicle treated samples determined by a two-tailed, paired *T* test. **E**, **F** 6 ×10^5^ Tgfβr2- and Ctrl-knockout *p18*^−/−^; *Brca1*^MGKO^ mammary tumor cells were inoculated into the left and right inguinal MFPs of NSG mice, respectively, in a pairwise manner. Two weeks after transplantation, mice were dissected. The volume (**E**) and expression of Vim (**F**) of the regenerated tumors were determined by measurement and IHC. Data in (**E**) represent the mean ± SD of four tumors in each group. The asterisks (*) denote a statistical significance from Tgfβr2- and Ctrl-knockout tumors determined by a two-tailed, paired *T* test. **G–I** 6 ×10^5^ Tgfβr2- or Ctrl-knockout *p18*^−/−^; *Brca1*^MGKO^ mammary tumor cells were inoculated into the MFPs of NSG mice. When newly generated tumors reached the maximum size allowed by IACUC in 4–7 weeks, or the mice became moribund, lungs were examined for gross appearance (**G**), H.E. staining (**H**), and quantification of the number of metastatic nodules (**I**). M, metastatic nodules. Data in (**I**) represent the mean ± SD for the numbers of metastatic nodules detected in all lobes of the lungs in each group (*n* = 4). Statistical significance was determined by a two-tailed, unpaired *T* test.

We knocked out Tgfβr2 in *p18*^*−/−*^*; Brca1*^*MGKO*^ tumor cells and found that Tgfβr2 KO led to lower expression of the EMT markers Vim, Fn, Snail, Zeb1, Slug, and higher expression of the epithelial markers including Cdh1 and Epcam (Fig. 5A, B). Morphologically, Tgfβr2-depleted tumor cells exhibited more epithelial-like phenotypes including islets of cells with close cell-cell contacts and cobblestone morphology, but less mesenchymal-like phenotypes including a reduction in spindle-shaped cells (Fig. 5C). We then treated cells with TGFβ, and found that in response to the treatment the elevation of Fn and Vim in control-depleted cells was drastically reduced in Tgfβr2-depleted cells (Figs. 5D, S6A). These results indicate that depletion of Tgfβr2 in *p18*^*−/−*^*; Brca1*^*MGKO*^ tumor cells inhibits EMT in vitro.

We transplanted tumor cells into mice. Though all four mice received 6 × 10^5^ Tgfβr2- and control-depleted *p18*^*−/−*^*; Brca1*^*MGKO*^ tumor cells developed tumors, tumors generated by Tgfβr2-knockout cells were significantly smaller than tumors generated by control cells (Fig. 5E). Tumors generated by Tgfβr2-depleted cells expressed noticeably reduced levels of Vim and p-Fra1 when compared with tumors generated by control cells (Figs. 5F, S6B). These results demonstrate that the deletion of Tgfβr2 in Brca1-deficient tumor cells inhibits EMT and suppresses tumorigenesis.

We also discovered that mammary tumors generated by Tgfbr2 KO cells produced significantly less metastatic nodules in the lung when compared with the tumors initiated by control cells (Fig. 5G-I). These results indicate that the deletion of Tgfβr2 inhibits the metastatic potential of Brca1 deficient mammary tumor cells.

### Inhibition of Tgfβr2 in Brca1 deficient tumor cells suppresses EMT and tumor initiation

**Fig. 6:**
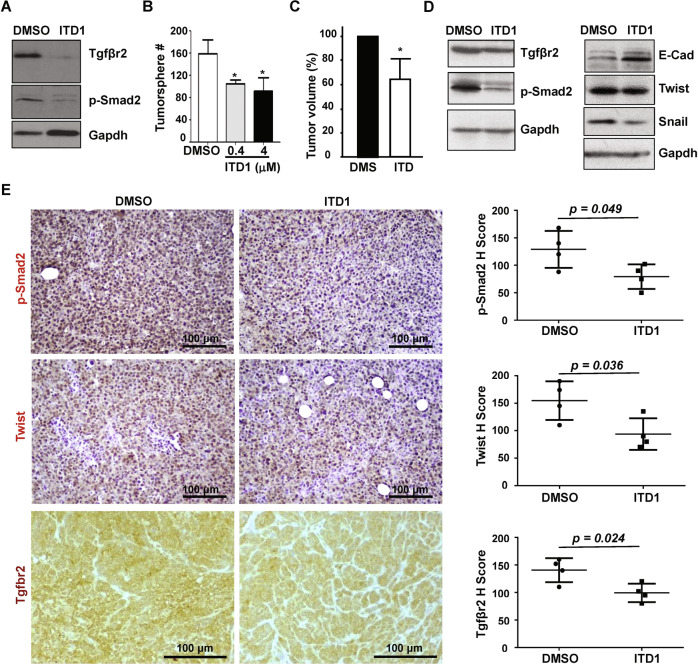
Pharmaceutical inhibition of Tgfβr2 in Brca1 deficient tumor cells suppresses EMT, tumorsphere formation, and tumor initiation. **A** *18*^−/−^; *Brca1*^*MGKO*^ tumor cells treated with ITD1 at 4 μm for 24 h were analyzed by western blot. **B** 10^4^ cells dissociated from*18*^−/−^; *Brca1*^*MGKO*^ primary tumorspheres were treated with ITD1. Secondary spheres formed after 6 days of treatment were counted from quadruplicate experiments. Data represent the mean ± SD from two independent primary tumorspheres. **C** 1000 *p18*^−/−^; *Brca1*^*MGKO*^ tumorsphere-dissociated cells pretreated with DMSO or ITD1 for 6 days were transplanted into MFPs of NSG mice. Four weeks later, mice were dissected and tumor volumes were measured. Values represent the average tumor volumes ± SD of four tumors. The asterisk (*) in (**B**) and (**C**) denotes a statistical significance from ITD1 treated and DMSO treated samples determined by a two-tailed, unpaired *T* test. **D**, **E** Representative tumors generated by DMSO- or ITD1-pretreated *p18*^−/−^; *Brca1*^*MGKO*^ cells were analyzed by Western blot (**D**) and IHC (**E**). The H-scores for p-Smad2, Twist, and Tgfβr2 in IHC were calculated (E, right panel). The results represent the mean ± SD of four individual tumors per group. Statistical significance was determined by a two-tailed, unpaired *T* test.

We treated *p18*^−/−^; *Brca1*^*MGKO*^ tumor cells with a TGFβR2 inhibitor ITD1, which specifically enhances degradation of Tgfβr2 [28]. We confirmed that ITD1 treatment reduced the level of Tgfβr2 and its downstream effector protein p-Smad2 (Fig. 6A). ITD1 treatment of the cells from primary *p18*^−/−^; *Brca1*^*MGKO*^ tumorspheres significantly reduced secondary tumorsphere formation (Fig. 6B). We transplanted tumorsphere-dissociated cells that were pretreated with ITD1 into mice. Pretreatment of *p18*^−/−^; *Brca1*^*MGKO*^ tumorsphere-dissociated cells with ITD1 led to significantly smaller tumors than those with vehicle pretreatment (Fig. 6C). Analysis of the newly generated tumors revealed that relative to control tumors, ITD1 tumors exhibited less Tgfβr2, p-Smad2, and EMT-TFs including Twist and Snail, but more E-Cad (Fig. 6D, E). These data suggest that pharmaceutical inhibition of Tgfβr2 in Brca1-deficient tumor cells suppresses EMT and their potential for tumor initiation.

### *BRCA1* and *TGFβR2* expression levels are inversely related in human breast cancers

**Fig. 7:**
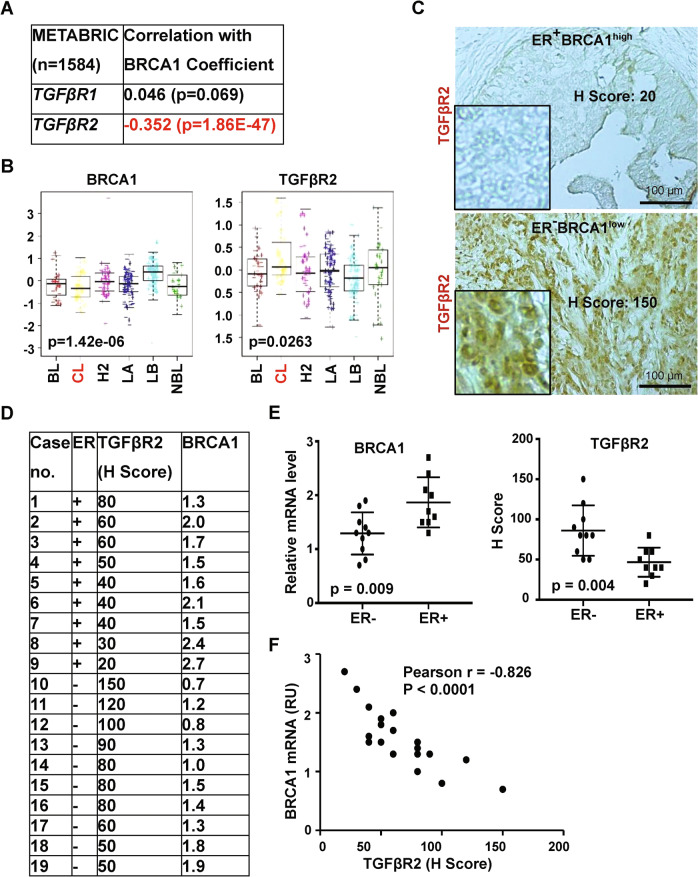
BRCA1 and TGFβR2 levels are inversely related to the CL subtype of human breast cancer. **A** Correlation analysis of the expression of *BRCA1* and TGFβR2 for MetaBric breast cancer patients. **B** Analysis of gene expression in NKI295 breast cancer patients according to tumor subtype. BL basal-like, CL claudin-low, H2 Her2-enriched, LA luminal A, LB luminal B, NBL normal breast-like. **C** Representative immunostaining of ER positive and negative invasive human breast cancers with antibodies against TGFβR2. **D, E** Summary of expression of TGFβR2 by IHC and *BRCA1* by Q-RT-PCR (**D**). The expression levels of TGFβR2 were quantified by H-scores. The expression of *BRCA1* was determined by Q-RT-PCR. *BRCA1* mRNA levels relative to that of T47D cells were determined as we previously reported [19]. Analysis of the expression of TGFβR2 by IHC and *BRCA1* by Q-RT-PCR in ER + and ER-invasive breast cancers (**E**). Statistical significance was determined by a two-tailed, unpaired *T* test. **F** Correlation analysis of BRCA1 mRNA levels and H scores of TGFβR2 expression for breast cancer samples.

Prompted by these observations in mouse models and prior studies showing enhanced expression of TGFβR2 in CSC-enriched BLBC cells [14, 15], we examined the relationship between *BRCA1* with *TGFβR2* mRNA levels in human breast cancer sample sets [3, 29, 30]. Just as in our mouse model, we observed a significantly inverse relationship between BRCA1 and *TGFβR2*, and a less significantly inverse relationship between BRCA1 and *TGFβR1* (Figs. 7A, S7B). Consistent with our observations in mouse models, human CL tumors show low BRCA1 mRNA and higher *TGFβR2* mRNA levels (Figs. 7B, S7A).

To test if these observations are consistent at the protein level we turned to our previously published resource of 43 invasive breast cancers [19]. We found that TGFβR2 H scores were significantly higher in ER-negative than ER-positive tumors, while BRCA1 mRNA was lower in ER-negative tumors. Importantly, across both ER-negative and ER-positive tumors, we observed a significant inverse relationship between TGFβR2 protein and BRCA1 mRNA levels (Figs. 7C–F, S7C). Together, these clinical findings are consistent with our results in mice, thereby suggesting an opportunity to use murine systems to further explore how BRCA1 status, TGFβR2 signaling, and EMT intersect to control the biology of human tumors.

We examined EMT, basal-like, and CL signatures in the TCGA breast cancer dataset [3, 31, 32]. Not accounting for intrinsic subtype, BRCA1 mutant tumors showed an elevation of EMT/CL-like features as compared to BRCA1 Wt tumors (Fig. S8A), although not to the same extent as CL tumors. Since none of the five CL tumors were BRCA1 mutant, we were unable to analyze the correlation between BRCA1 mutation status and CL features in this subtype. Thus, we next limited our analysis to basal-like breast cancers, and again found a tendency for enhanced EMT/CL-like features in BRCA1 basal-like tumors, however, these differences were not statistically significant when compared to BRCA1 Wt tumors. We speculate that these observations might indicate that loss of BRCA1 leads to more EMT-like features in a proportion of cells within a tumor, but not the extent that the intrinsic subtype of the tumor bulk is significantly changed. In agreement, we observed that the basal subtype expression signature in BRCA1 mutant basal-like tumors also trended lower than BRCA1 Wt basal-like tumors (Fig. S8B).

## Discussion

A number of studies have reported a relationship between BRCA1 and the basal-like TNBC subtype [2, 33, 34]. Here we expand on these studies by showing Brca1 alters the expression of Tgfβr2 to elevate Tgfβ signaling and EMT in breast cancer cells. Targeted deletion of, or pharmaceutical inhibition of, Tgfβr2 in Brca1-deficient tumor cells reduced EMT and suppressed tumorigenesis and metastasis. Moreover, we found BRCA1 mRNA was low and TGFβR2 mRNA was high in the claudin-low TNBC subtype. Thus, BRCA1 function may not only be implicated in the basal-like molecular subtype but also in claudin-low as well. Importantly, this also builds upon the body of work showing a correlation between EMT and the CL subtype [4, 35, 36] by showing a mechanistic connection between BRCA1 and TGFβ signaling in an epithelium-autonomous manner.

While our study demonstrates TGFβR2 induces malignant phenotypes in p18/BRCA1-deficient tumors, other studies show opposing roles in tumor suppression or tumor promotion [12, 14]. In favor of the malignant role for TGFβR2, overexpression of TGFβR2 or upregulation of TGFβ signature genes is associated with lung metastasis as well as lower survival in human BLBCs [16, 17], and higher stromal TGFβR2 is associated with poorer prognosis breast cancers [37]. Inhibition of TGFβR2 in human or mouse breast cancer cell lines reduces metastasis and blocks chemotherapy-induced CSC expansion [14, 38]. In support of the tumor suppressor function of TGFβR2, it has been found that in humans, reduction of or loss of TGFβR2 is associated with an increased risk of developing high-grade carcinoma in situ and invasive breast cancer [39, 40]. In addition, loss of TGFβR2 in stromal cancer-associated fibroblasts is linked to shortened patient survival [41]. In mouse models, the deletion of Tgfβr2, or expression of dominant-negative type Tgfβr2, results in or accelerates mammary tumor development and progression [14]. Collectively, these prior data and our study suggest that the role of Tgfβr2 is context-dependent and possibly related to BRCA1 function/regulation.

Understanding the genomic context contributing to Tgfβr2 expression and function could be important for guiding therapies. In particular, our study suggests that a targetable TGFβR signaling pathway exists in many BRCA1-deficient TNBC. Very few therapeutic options are available for ER-negative BLBCs. More than half of BLBCs have a dysfunctional BRCA1 pathway and harbor defects in DNA damage repair which make these patients initially respond well to DNA-damaging agents [2, 34, 42]. However, tumor recurrence and acquired resistance to DNA-damaging agents combine to decrease the survival of such patients [43, 44]. In addition, most BRCA1-deficient BLBCs carry a dysfunctional INK4-RB pathway [2, 45–47]. Specifically, our combination of p18 and Brca1 loss model those tumors with INK4-RB pathway deficits. Therefore, our results might suggest a context where Tgfβr2 might contribute to malignancy in human TNBC and reveal, for the first time, that a targetable TGFβR signaling pathway is directly activated by BRCA1-deficiency in the induction of EMT in breast cancers.

While this is all very straightforward in our controlled in vitro and mouse model systems, the manifestation in human tumors is more complex. In the limited number of CL tumors examined, which have molecular similarities to EMT [35], patterns of BRCA1 and TGFβR2 matched our experimental data in mouse models and human cell lines. Given that none of these tumors was BRCA1 mutant, these processes might also be epigenetically influenced. Further, our analysis of human basal-like tumors suggests that BRCA1 mutations might impact the proportion of cells with EMT-like features as there appears to be a trade-off between “basal-ness” and “claudin-low-ness” tracking with BRCA1 status. This may imply that additional cues are required to drive EMT in these cells, which we anticipate might include local production of TGFβ; additional experimental studies will be needed to confirm this.

The above observations highlight our incomplete understanding of the mechanisms leading to EMT and claudin-low characteristics in TNBC. Yet, with the parallels between our mouse models and human tumors (and cell lines), we anticipate continued progress using this murine system to understand TNBC tumor heterogeneity. In addition to carrying a dysfunctional INK4-RB pathway, most BRCA1-deficient BLBCs harbor p53 mutations [2, 45–47]. Most studies of *Brca1* in mice co-mutate *Brca1* with one of the genes in the *p53* pathway [48]. However, mutation of *p53* alone stimulates TGFβR2 and induces basal-like and CL subtype mammary tumors [4, 49, 50]. Our results demonstrate that Brca1 suppresses TGFβR2-mediated EMT in mammary tumorigenesis in the context of INK-RB inactivation under a genetically intact *p53* background. Further, we find that some of the Brca1 deficient mammary tumors are CL [3, 33, 51] and that Brca1 deficiency induces basal-like mammary tumors with activation of EMT [18, 19], which is recently confirmed by an independent group [52, 53]. Thus, we provide an important mouse model for specific subsets of TNBC and uncover key molecular alterations that govern tumor heterogeneity and the malignant potential of these tumor types that might be effectively managed by targeting the TGFβ pathway.

## Materials and methods

### Mice, histopathology, and immunostaining

The generation of *p18*^*−/−*^*, p18*^*+/−*^*, and p18*^*−/−*^*:Brca1*^*+/−*^ mice in Balb/c background, and *p18*^*−/−*^ *and p18*^*−/−*^*;Brca1*^*f/f*^; MMTV-Cre (*p18*^*−/−*^*; Brca1*^*MGKO*^) mice in Balb/c-B6 mixed background has been previously described [18, 19, 54]. The Institutional Animal Care and Use Committee at the University of Miami and Shenzhen University approved all animal procedures. Animals were housed in a specific pathogen-free environment with a 12/12 light cycle. Animals were euthanized by exposure to isoflurane followed by cervical dislocation. At least four female mice were analyzed for each genotype, or were transplanted with each type of tumor cells. Histopathology and immunohistochemistry (IHC) were performed as previously described [18, 19, 54]. The primary antibodies used were: TGFβR1, TGFβR2 (Santa Cruz), p-Smad2, p-FRA1 (Cell signaling), CK5 (Covance), and Vim (Abcam). Immunocomplexes were detected using the Vectastain ABC DAB kit according to the manufacturer’s instructions (Vector Laboratories). The positive results of IHC were quantified by H-score, as previously described [55].

### Mammary tumor cell preparation, cell culture, tumorsphere formation assay, overexpression and knockdown of BRCA1, and TGFβ treatment

Primary mammary tumors were dissected and cell suspensions were prepared as previously described [18, 19, 54]. T47D, HCC1937, SUM149, MDA-MB-231, and MCF7 cells were cultured per ATCC recommendations. For primary tumorsphere formation assay, mammary tumor cells were plated onto ultra-low attachment plates, in serum-free DMEM-F12, as previously described [19, 54]. Primary tumorspheres formed were collected and counted after 10 days of culture. For the secondary tumorsphere formation assay, primary tumorspheres formed were collected and dissociated. 10^4^ dissociated cells were plated in triplicates with or without ITD1 (BioVision) treatment. Secondary tumorspheres that formed after 6 days of culture were counted. For ectopic expression of BRCA1, HCC1937 and SUM149 cells were transfected with pBabe-empty or pBabe-HA-BRCA1 as previously described [19]. For BRCA1 knockdown, T47D, MCF7, and MDA-MB-231 cells were infected with pGIPZ-empty (Sh-Ctrl) and pGIPZ-shBRCA1 (Sh-BRCA1) lentiviral vectors that were purchased from Open Biosystems, as previously described [19]. For TGFβ treatment, cells were starved in a serum-free medium for 24 h and then exposed to DMSO or TGFβ with an indicated period before analysis.

### CRISPR-mediated Tgfβr2 knockout, transplantation, and analysis of metastasis

For CRISPR-mediated Tgfβr2 knockout in primary tumor cells, Tgfβr2 Double Nickase and control Double Nickase plasmids (Santa Cruz) were transfected into *p18*^*−/−*^*; Brca1*^*MGKO*^ primary tumor cells, respectively, following the manufacturer’s protocol. After selection with puromycin for 3 days, GFP-positive cells were FACS sorted for further analysis. For in vivo transplantation, primary *p18*^*−/−*^*; Brca1*^*MGKO*^ and *p18*^*−/−*^ or Tgfβr2- and control-depleted *p18*^*−/−*^*; Brca1*^*MGKO*^ tumor cells were suspended in a 50% solution of Matrigel (BD) and then inoculated into the left and right inguinal mammary fat pads (MFPs) of 4-week-old female NSG mice (Jackson Laboratory), respectively, in a pairwise manner. Four or two weeks after transplantation, animals were euthanized and mammary tumors were analyzed. For ex vivo transplantation, primary *p18*^−/−^; *Brca1*^MGKO^ tumor cells were cultured to generate primary tumorspheres. 10^4^ cells dissociated from primary tumorspheres were treated with DMSO or ITD1 for 6 days. 1,000 live cells were transplanted into MFPs of NSG mice. Four weeks after transplantation, animals were euthanized and mammary tumors were analyzed. The tumor size was measured daily with a caliper. Tumor volumes were calculated as V = a × b^2^/2, while “a” is the largest diameter and “b” is the smallest.

For analysis of lung metastasis from mammary tumors, *p18*^*−/−*^*; Brca1*^*MGKO*^ tumor cells were inoculated into the MFPs of NSG mice. When newly generated tumors either reached the IACUC designated endpoint size (1.3 cm^3^; in 4–7 weeks) or the mice became moribund, the lungs were examined for detection of metastasis. For quantification of the number of metastatic nodules in the lungs, fixed lung tissues of all five lobes were sagittally sectioned at 200-μm intervals. At least three sections for each lobe were prepared and stained with H.E. The metastatic nodules in each lobe of lung tissue were confirmed by H.E. staining, counted under a microscope, and averaged. The number of nodules in all lobes was then calculated.

### Microarray analysis, western blot, and chromatin-immunoprecipitation (ChIP) assay

RNA was extracted and purified from tumors using a RNeasy kit (Qiagen). Tumor RNA was reverse transcribed, amplified, and labeled with Cy5. Wt mammary tissue reference RNA was reverse transcribed, amplified, and labeled with Cy3. The amplified sample and reference were co-hybridized to Agilent 4x180k custom mouse microarrays and were analyzed as previously described [51]. Gene expression data for *p18*^*−/−*^*, p18*^*+/−*^*, and p18*^*−/−*^*: Brca1*^*+/−*^ tumors was uploaded to the UNC microarray database. The Log(base2) of R/G Lowess Normalized Ratio (Mean) was taken for each array. For each channel, Lowess Normalized Net (Mean) greater than or equal to 10 was used to determine the presence of a signal. Genes present across 70% of samples were maintained and the remaining missing values were calculated and replaced using KNN imputation. Next genes were median centered and samples were standardized for the working gene matrix. Gene set enrichment analysis (GSEA) was performed using the GenePattern server [56, 57]. The gene signatures for Tgfβ and NF-КB signaling activities and EMT were published [35, 58, 59]. Gene expression data of mammary tumors coming from *p18*^−/−^, *p18*^*+/−*^, and *p18*^−/−^;*Brca1*^+/−^ mice were deposited on the Gene Expression Omnibus under accession GSE155239. Data were obtained using Agilent microarrays as published [60]. Data were combined with a published dataset containing K14-Cre;p53^f/f^;Brca1^f/f^ mouse mammary tumors as well as additional intrinsically credentialed genetically engineered mouse models (GEMMs) using the UNC Microarray database [33]. Intensity values were lowess normalized and relative counts established using median centering.

For the western blot, tissue and cell lysates were prepared as previously reported [19, 54]. The primary antibodies used were: BRCA1 and TGFβR2, (Santa Cruz), Vimentin, E-cad, Snail, Twist, p-Fra1, p-Smad2, p-Erk, p-Jnk, p-Akt (Cell signaling), and Gapdh (Ambion). All blots were derived from the same experiment and were processed in parallel. ChIP assays were carried out as previously described [19, 54]. Briefly, cells were treated with 1.5% formaldehyde and sonicated. Anti-BRCA1 antibody (D-9, Santa Cruz) or control mouse IgG was used to precipitate chromatin associated with BRCA1. Q-PCR was performed to determine the relative abundance of target DNA. Specific primers for the analysis of BRCA1 binding to TGFβR2 are listed in Table. S1.

### Dual-luciferase reporter assay

The human TGFβR2 promoter region -2589 to -1613 that covers P5 and part of P4 primers used for ChIP analysis and contains a GATA3/BRCA1 binding site with consensus sequences, TGATTG, at -2115 was inserted into the pGL3-basic (Promega), as pGL3-TGFβR2-WT. With pGL3-TGFβR2-WT as a template, GATA3 binding site TGATTG was mutated into TACTTG to generate pGL3-TGFβR2-Mut by site-directed mutagenesis. Renilla plasmid was a gift from S. Y. Fuchs (University of Pennsylvania). For the TGFβR2 promoter-luciferase reporter assay, T47D-sh-Ctrl, T47D-sh-BRCA1, and SUM149 cells were seeded in a 24-well plate. T47D-sh-Ctrl and T47D-sh-BRCA1 cells were transfected with Renilla and either pGL3-TGFβR2-WT or pGL3-TGFβR2-Mut. SUM149 cells were transfected with Renilla plus pGL3-TGFβR2-WT or pGL3-TGFβR2-Mut, as well as pBabe-empty or pBabe-HA-BRCA1. The transfection efficiency was monitored using the Renilla vector as an internal control. Two days after transfection, cell lysates were collected and subjected to luciferase assay using the Dual-Luciferase Reporter Assay System (Promega). Two independent transfection experiments were conducted, and each luciferase assay was performed in triplicates. Normalized data was calculated as the ratio of the firefly/Renilla luciferase activities.

### Human tumor samples and gene-expression data sets

Formalin-fixed paraffin-embedded (FFPE) human breast cancer samples lacking patient-identifying information were obtained from the Tissue Bank Core Facility at the University of Miami and the Department of Pathology at Shenzhen University. Samples used for this study consisted of non-treated invasive breast carcinomas with known ER status. Tumor samples were microdissected. Samples with tumor cell content >75% were used for RNA extraction. The expression of *BRCA1* was determined by Q-RT-PCR, as previously reported [19]. Pearson correlation coefficient and *P* value (two-tailed) of BRCA1 mRNA levels and H scores of TGFβR2 expression for breast cancer samples were calculated by GraphPad Prism software. The correlation of expression of *TGFβR1/2* mRNA with *BRCA1* mRNA was analyzed with NKI295 [29], MetaBric [30], and UNC337 [3] human breast cancer datasets. The NKI295 dataset was analyzed to compare gene expression versus six breast cancer subtypes using a two-way analysis of variance (ANOVA).

### Statistical analysis

All data are presented as the mean ± SD for at least three repeated individual experiments for each group. Quantitative results were analyzed by two tailed Fisher Exact test or two-tailed Student’s *t*-test. *P* < 0.05 was considered statistically significant.

Pearson correlation coefficient of BRCA1 and TGFβR2 expression was calculated by GraphPad Prism software. The NKI295 dataset was analyzed to compare gene expression versus six breast cancer subtypes using a two-way analysis of variance (ANOVA).

## Supplementary information

Table S1 and Figure S1-S9

Agreement emails to authorship change

Checklist

## Data Availability

Murine tumor gene expression data was deposited at the Gene Expression Omnibus under accession number GSE155239. All other datasets can be found as part of this manuscript.
