# Supplementary material for: Loss of function of BRCA1 promotes EMT in mammary tumors through activation of TGFβR2 signaling pathway
Source: Cell Death Dis. 2022 Mar 2;13(3):195. doi: 10.1038/s41419-022-04646-7 (PMC8891277; doi:10.1038/s41419-022-04646-7)

Table S1

Primer sequence for accessing the occupancy of BRCA1 on the TGFβR2 locus

Primer 1	FW<br>RV	GGAACTCCTGAGTGGTGTGG<br>AGCCCCTAGCTCTCTCGTAG	Site -38 (-120~-101)<br>(-28~-9)
Primer 2	FW<br>RV	GAGGCGGCAGATGTTCTGAT<br>CAGGAGTTCCTCGCTCCAAG	Site -181 (-224~-205)<br>(-130~-111)
Primer 3	FW<br>RV	GACCAGAGGGCTGTACAGAAT<br>GCCCAAGACATTCCTGGTGTA	Site -956 (-1023~-1003)<br>(-896~-876)
Primer 4	FW<br>RV	TTGGAGGCTTGAAGACACGTT<br>AGTTTCTCAAACGAGAGCTGA	Sites -1598, -1587, -1551 (-1633~-1613)<br>(-1504~-1484)
Primer 5	FW<br>RV	CGGTAACAATGTTTTCTCCAGAGT<br>TTATGTATCTTTCCCTCCACTTACT	Site -2115 (-2155~-2132)<br>(-2010~-1986)
Primer 6	FW<br>RV	TTTGCAAATGGCCCAGTTTTT<br>TGTTGAAAGGGATGTCTGATACC	Site -3039 (-3143~-3123)<br>(-3031~-3009)
Primer 7	FW<br>RV	AGGGTGGATGACCCTCACAA<br>CCAGCCTCATTCTCAGCTCTTA	Site -3494 (-3573~-3554)<br>(-3491~-3470)
Primer 8	FW<br>RV	TGAAGACAAGAAACACACACTGA<br>ACCCTAATGATTGTAAAGCCCT	Sites -3842, -3839, -3772 (-3869~-3847)<br>(-3781~-3760)

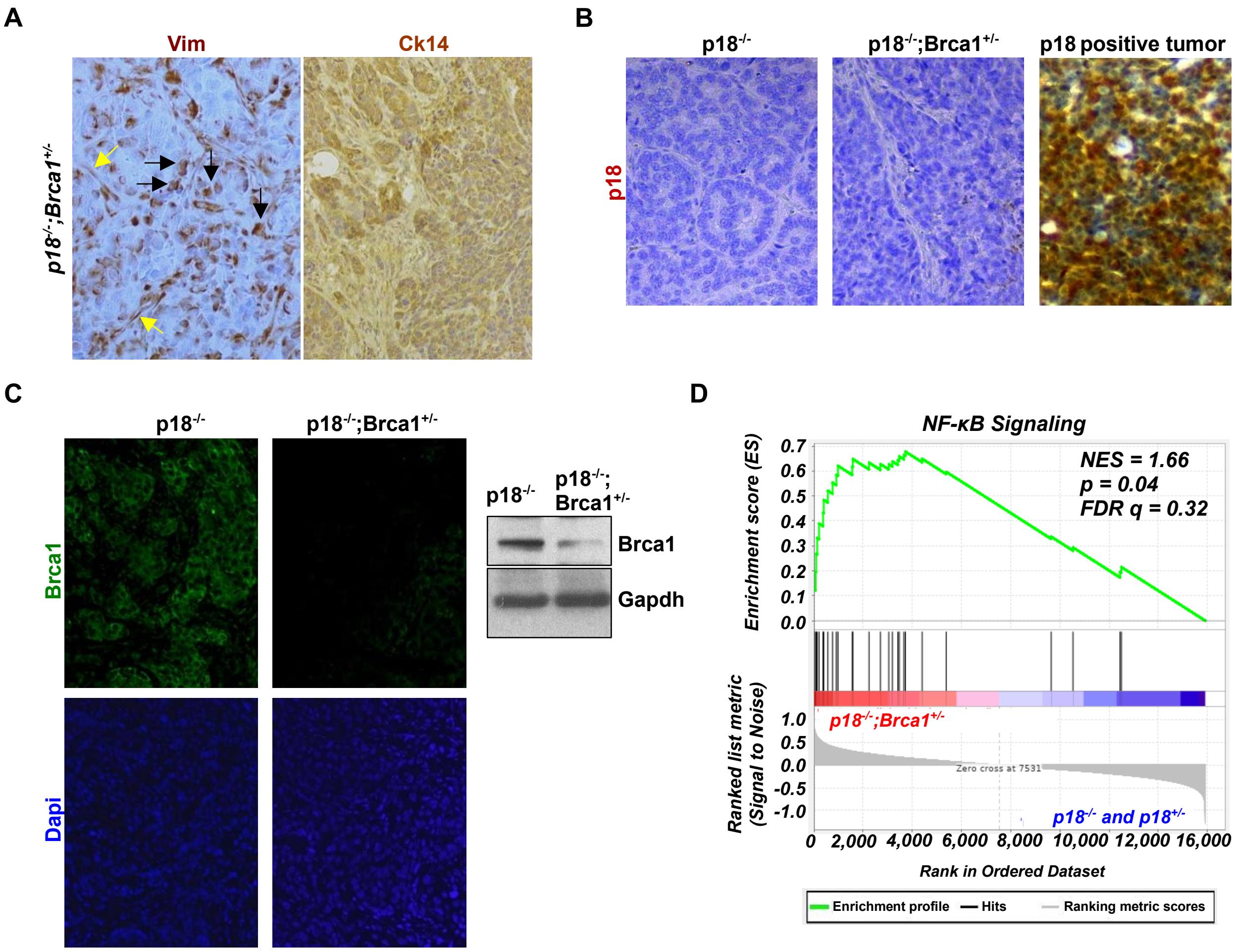

Fig. S1. Analysis of spontaneous mouse mammary tumors. (A) serial sections from a *p18*$^{-/-}$;*Brca1*$^{+/-}$ tumor were immunostained with antibodies against Vim and Ck14. Vim positive tumor cells (Black arrows) and stromal cells (Yellow arrows) are indicated. (B) IHC analysis of p18 expression in representative *p18*$^{-/-}$;*Brca1*$^{+/-}$ and *p18*$^{-/-}$ tumors. Mammary tumor generated by transplantation of Gata3 deficient MMTV-PyMT cells was used as positive control for p18 staining, as we previously reported (Bai F, Theranostics, 2022). (C) Analysis of Brca1 expression in representative *p18*$^{-/-}$;*Brca1*$^{+/-}$ and *p18*$^{-/-}$ tumors by immunofluorescent staining (left) and western blot (right). (D) GSEA enrichment plot for a signature for NF-KB signaling activity (Schon, JNCI, 2008). Note, heterozygous germline deletion of Brca1 in p18 deficient mice activates NF-KB signaling in mammary tumor cells. NES: Normalized Enrichment Score (NES), Nominal p-value (p), and False Discovery Rate q-value (FDR q) were detected comparing *p18*$^{-/-}$;*Brca1*$^{+/-}$ tumors (n=10) to *p18*$^{-/-}$ and *p18*$^{+/-}$ tumors (n= 10).

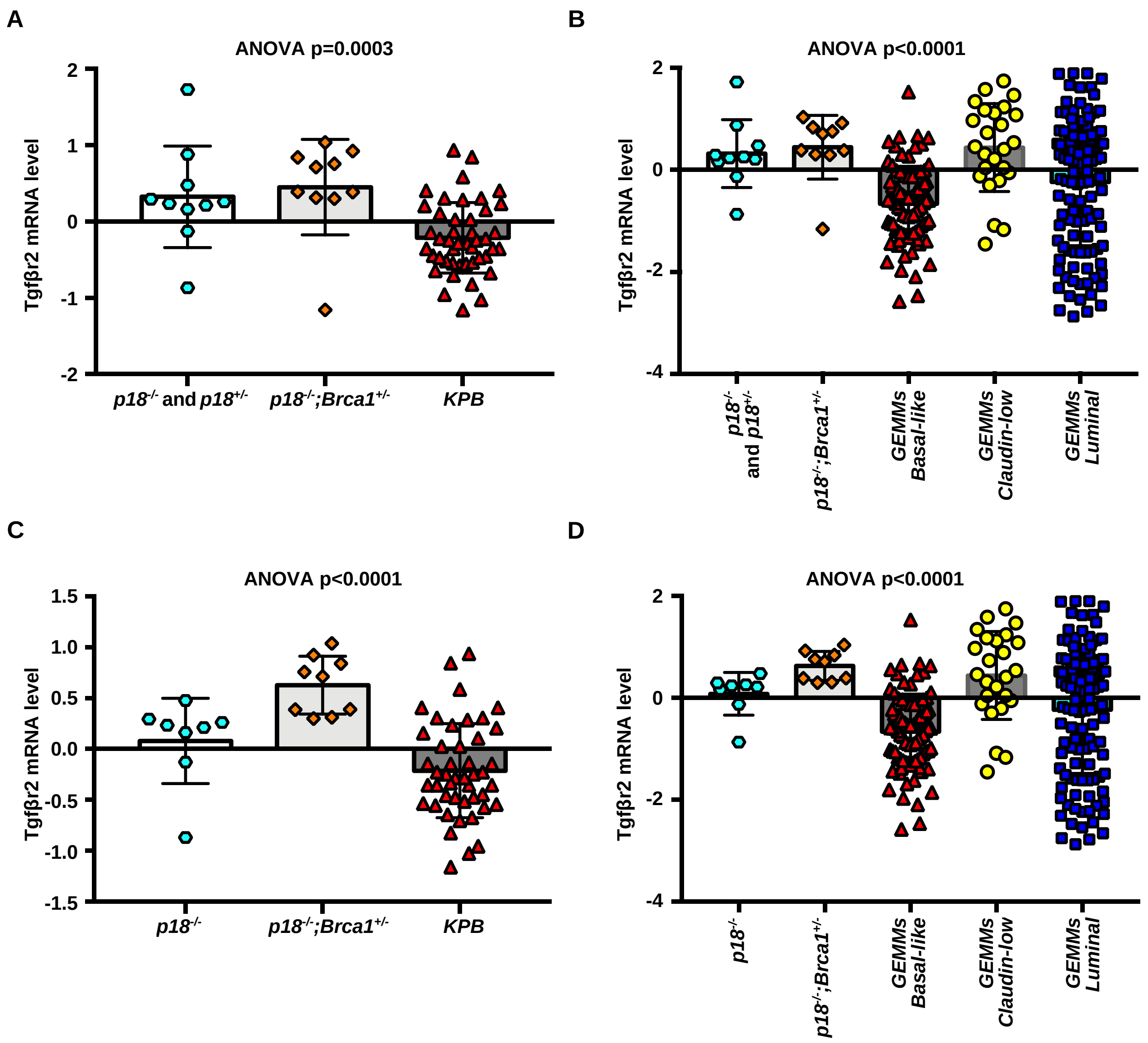

Fig. S2. Analysis of Tgfβr2 mRNA levels in mammary tumors derived from various mouse models by microarray. (A, B) Analysis of non-filtered tumor samples. $p18^{-/-}$ and $p18^{+/-}$ samples (n = 10) including nine $p18^{-/-}$ and one $p18^{+/-}$ samples and $p18^{-/-}$;$Brca1^{+/-}$ samples (n = 10) were analyzed. KPB, K14-Cre;$p53^{f/f}$;$Brca1^{f/f}$. GEMMs, genetically engineered mouse models. (C, D) Analysis of an outlier removed $p18^{-/-}$;$Brca1^{+/-}$ (n = 9) and an outlier removed $p18^{-/-}$ (n = 8) samples, as well as other tumor samples. Note the high levels of Tgfβr2 in $p18^{-/-}$;$Brca1^{+/-}$ and claudin-low subtype tumors from GEMMs in (D). Also note the minimal expression level of Tgfβr2 in KPB mammary tumors in (C).

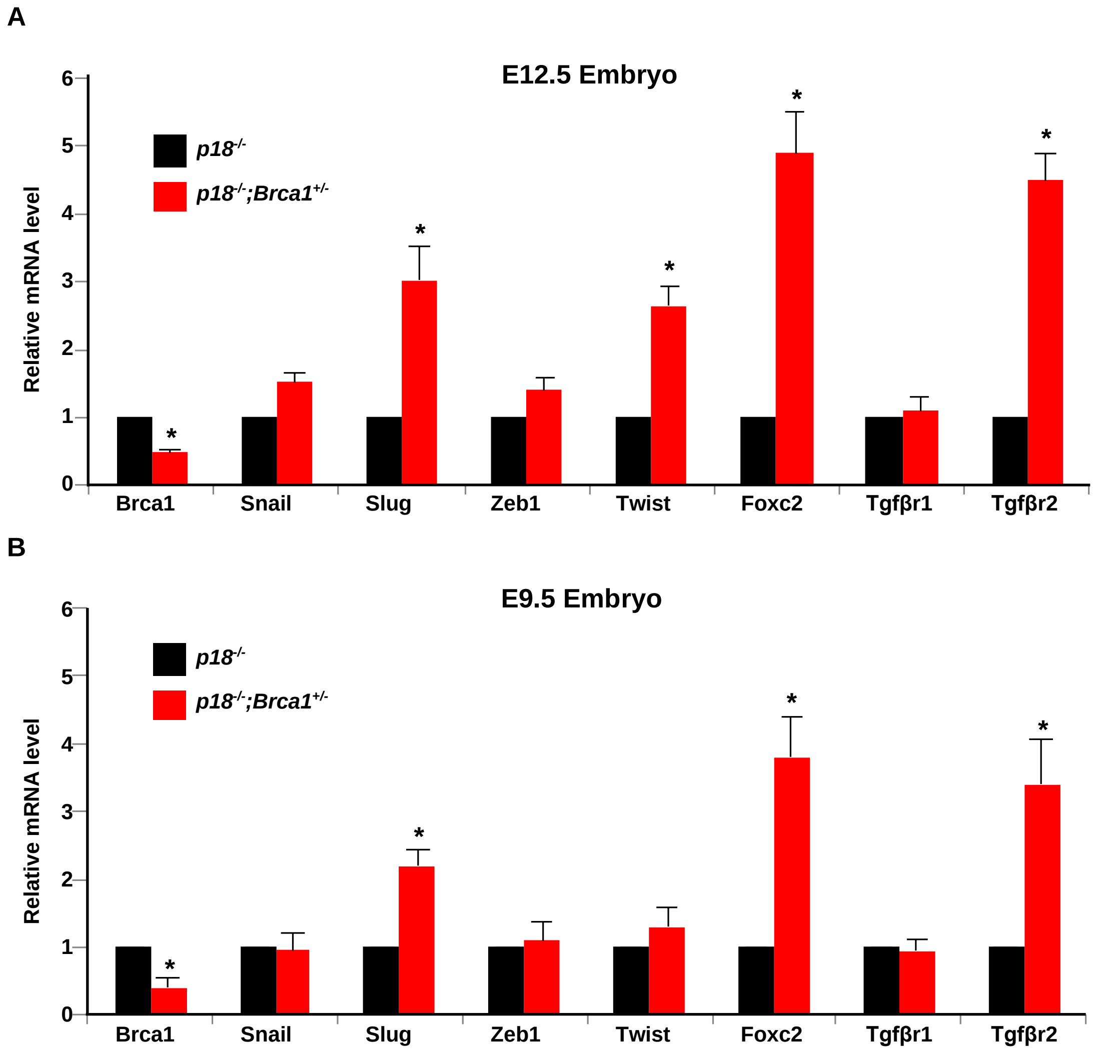

Fig. S3. Brca1 deficiency enhances the expression of Tgfbr2 and EMT-TFs in embryos. (A, B) mRNA levels of the indicated genes in *p18*$^{-/-}$ and *p18*$^{-/-}$;*Brca1*$^{+/-}$ embryos at E12.5 (A) and E9.5 (B) were analyzed by q-RT-PCR. Data represent the mean ± SD from triplicates of each of the two independent embryos. The asterisk (*) denotes a statistical significance from *p18*$^{-/-}$ and *p18*$^{-/-}$;*Brca1*$^{+/-}$ embryos determined by a two-tailed, unpaired T test.

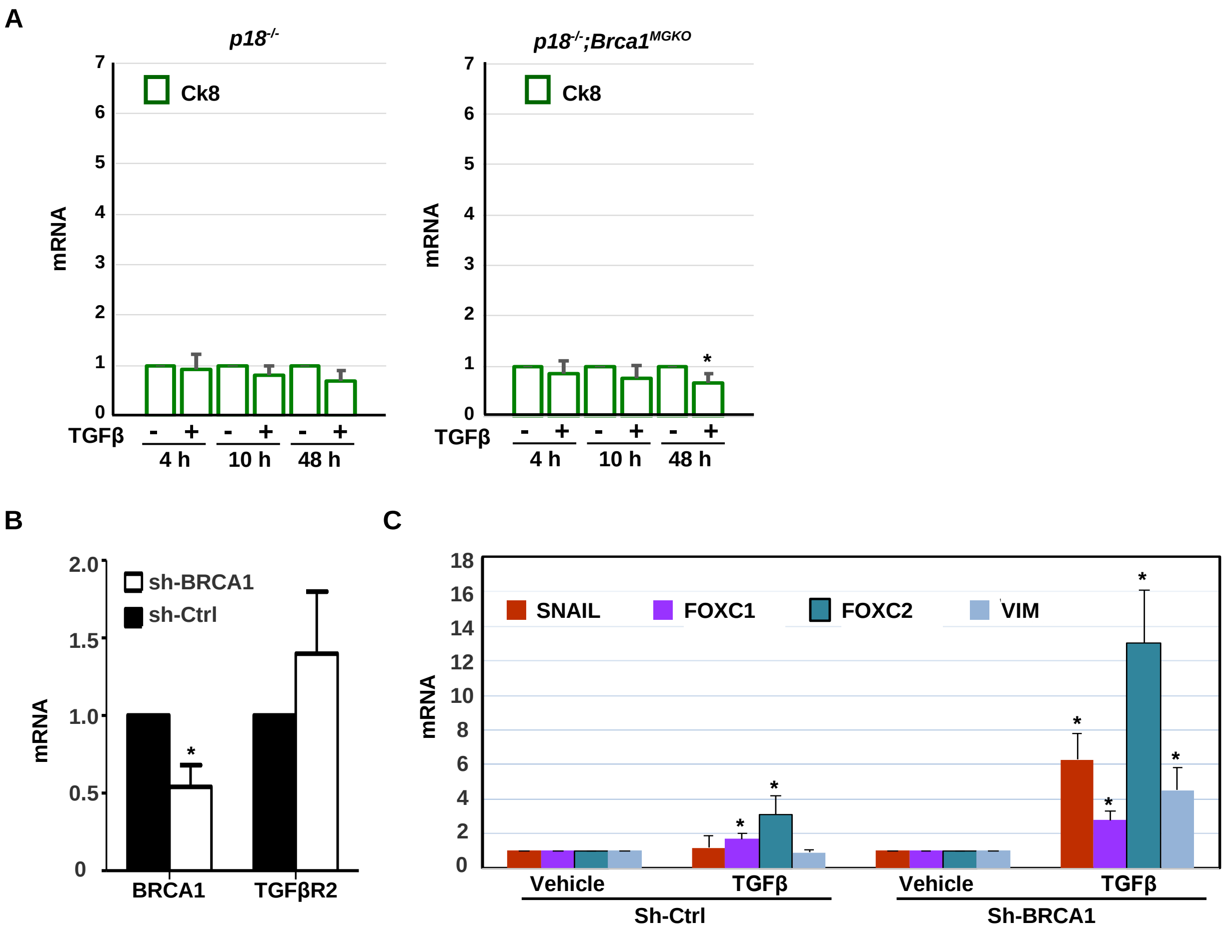

Fig. S4. BRCA1 depletion sensitizes tumor cells to TGFβ-mediated EMT activation. (A) *p18*$^{-/-}$ and *p18*$^{-/-}$; *Brca1*^{MGKO} mammary tumor cells were treated with vehicle or TGFβ for different time periods, and then analyzed by qRT-PCR. (B) mRNA levels in T47D-sh-Control (Ctrl) and T47D-sh-BRCA1 cells were determined. Data represent the mean ± SD from triplicates of each of the two independent experiments. (C) mRNA levels in T47D-sh-Ctrl and T47D-sh-BRCA1 cells treated with TGFβ for 24 hours were analyzed. Data represent the mean ± SD from duplicates of two independent experiments. The asterisk (*) denotes a statistical significance from TGFβ- and vehicle-treated samples (A, C) or sh-BRCA1 and sh-Ctrl samples (B) determined by a two-tailed, paired T test.

*

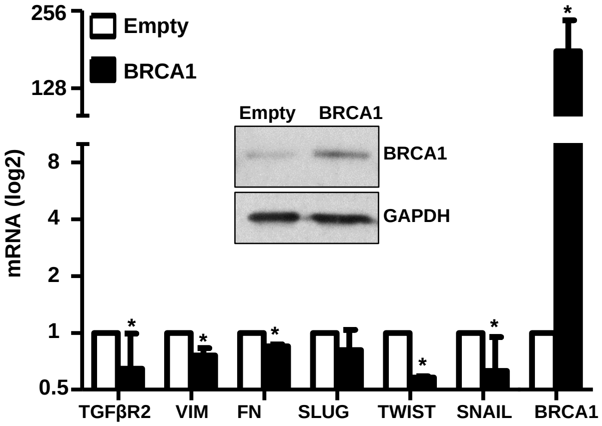

Fig. S5. BRCA1 represses transcription of TGFβR2 and EMT-associated genes. HCC1937 cells were transfected with pBabe-empty (Empty) or pBabe-HA-BRCA1 (BRCA1). Expression of genes indicated in HCC1937 cells were determined by western blot and qRT-PCR. Data represent the mean ± SD from triplicates of two independent experiments. The asterisk (*) denotes a statistical significance from empty- and BRCA1-expressing samples determined by a two-tailed, paired T test.

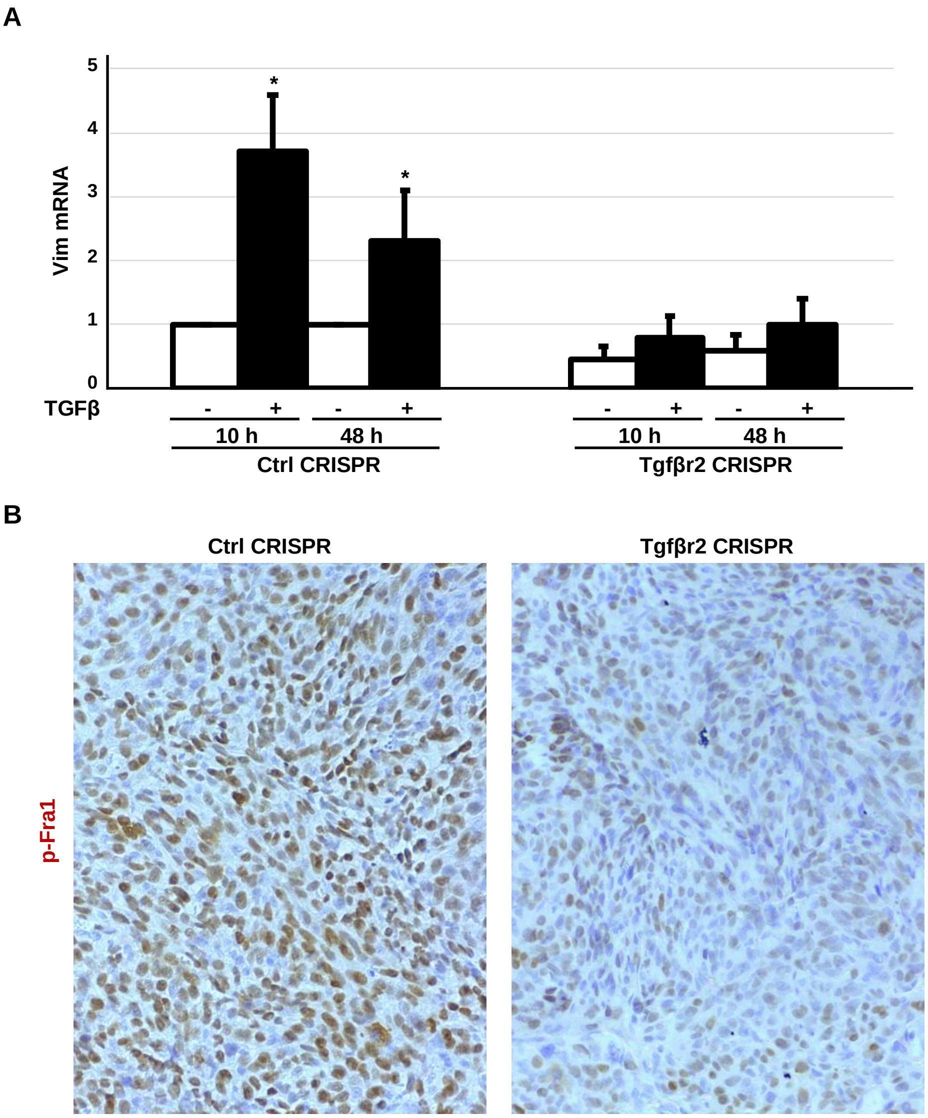

Fig. S6. Deletion of Tgfβr2 in Brca1-deficinet tumor cells inhibits EMT. (A) Tgfβr2- and Ctrl-knockout p18$^{-/-}$; Brca1^{MGKO} mammary tumor cells were treated with vehicle or TGFβ for different time periods, and the expression of Vim was then determined. Data represent the mean ± SD from duplicates of two independent experiments. The asterisk (*) denotes a statistical significance from TGFβ- and vehicle-treated samples determined by a two-tailed, paired T test. (B) Representative immunostaining of mammary tumors generated by Tgfβr2- (Tgfβr2 CRISPR) or Ctrl (Ctrl CRISPR)-depleted *p18*$^{-/-}$;*Brca1*^{MGKO} tumor cells with antibody against p-Fra1.

A

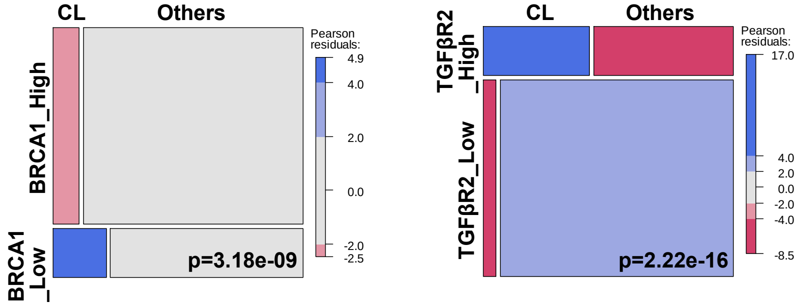

B

UNC337 (n=337)	Correlation with BRCA1 Coefficient
TGFβR1	-0.109 (p=0.045)
TGFβR2	-0.268 (p=6.12E-07)

NKI295 (n=295)	Correlation with BRCA1 Coefficient
TGFβR1	-0.133 (p=0.022)
TGFβR2	-0.245 (p=2.0E-05)

C

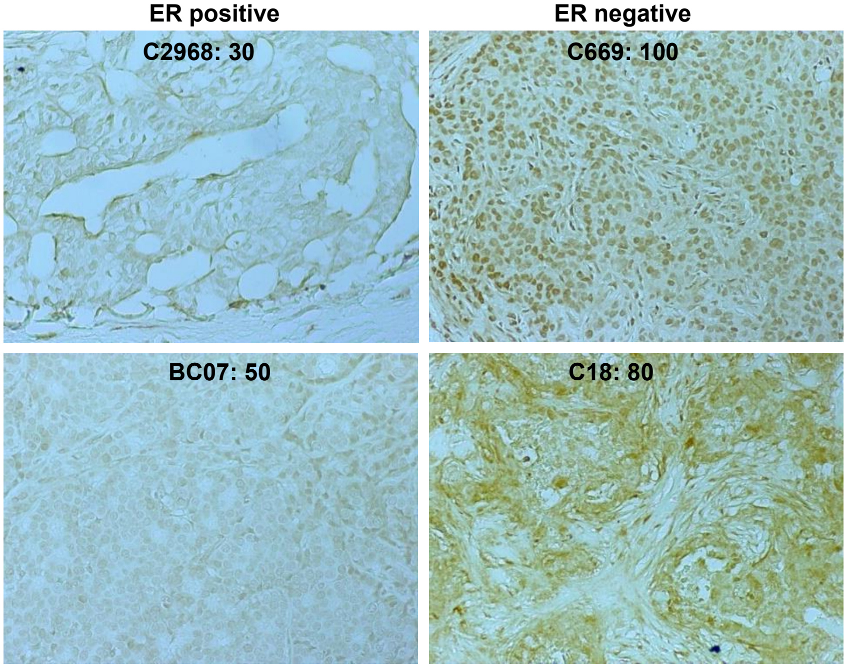

Fig. S7. Analysis of BRCA1 and TGFβR1/2 in human breast cancers. (A) Correlation analysis of BRCA1 and TGFβR2 mRNA levels with CL subtype of breast cancers in MetaBric breast cancer patients. Note the significant correlation of BRCA1 Low and TGFβR2 High with CL subtype of breast cancers. Others, all other subtypes; Blue color, over-represented; Red color, under-represented. (B) Correlation analysis of the expression of BRCA1 and TGFβR1/2 in UNC337 and NKI295 breast cancer patients. (C) Representative immunostaining of ER positive and negative invasive human breast cancers with antibody against TGFβR2. H-scores for TGFβR2 expression are shown.

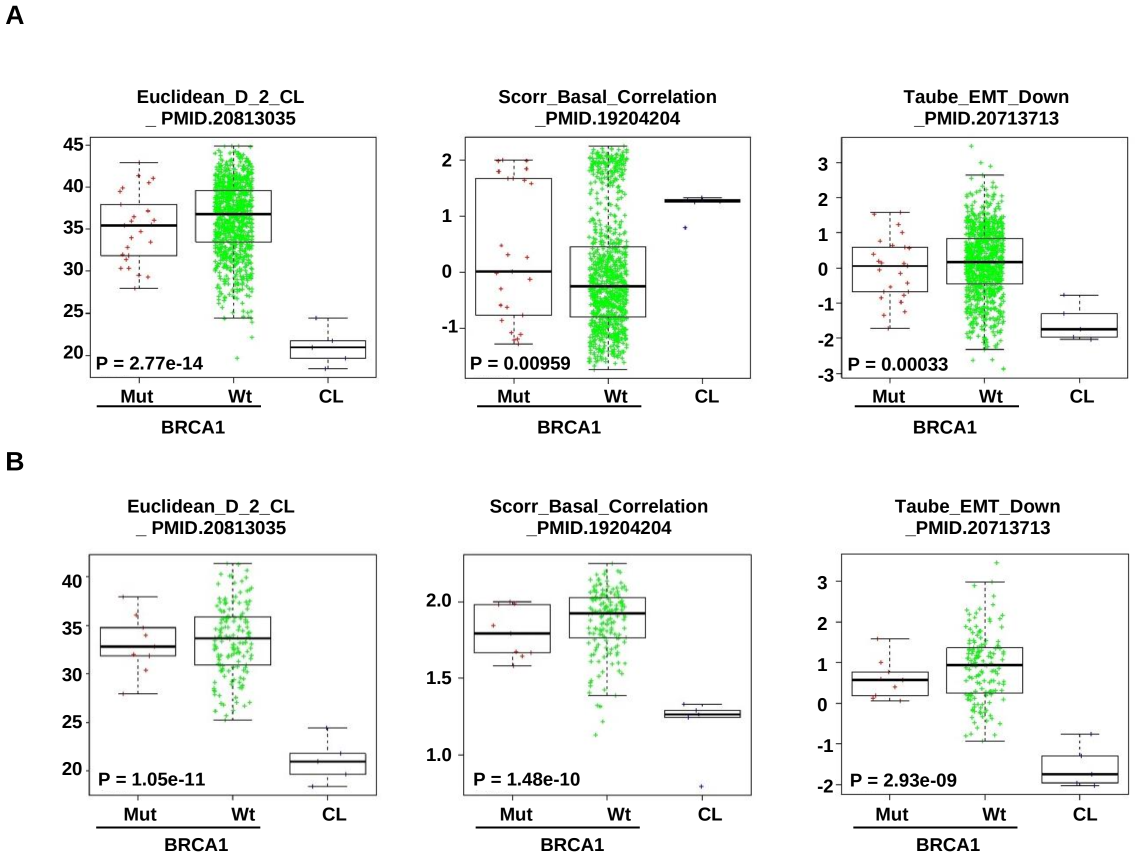

Fig. S8. Analysis of claudin-low (CL), basal, and EMT down signatures in human breast cancers (A) or in basal-like human breast cancers (B) according to the status of BRCA1 gene in the published breast cancer datasets (Prat A., Breast Cancer Res. 2010; Parker JS, JCO, 2009; Taube JH, PNAS, 2010). Mut, mutant; Wt, Wild type.

Fig. S9. Raw data for the blots included in each figure and supplementary data.

For Fig. 2B

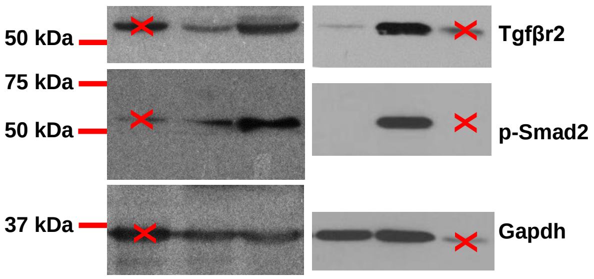

For Fig. 2C

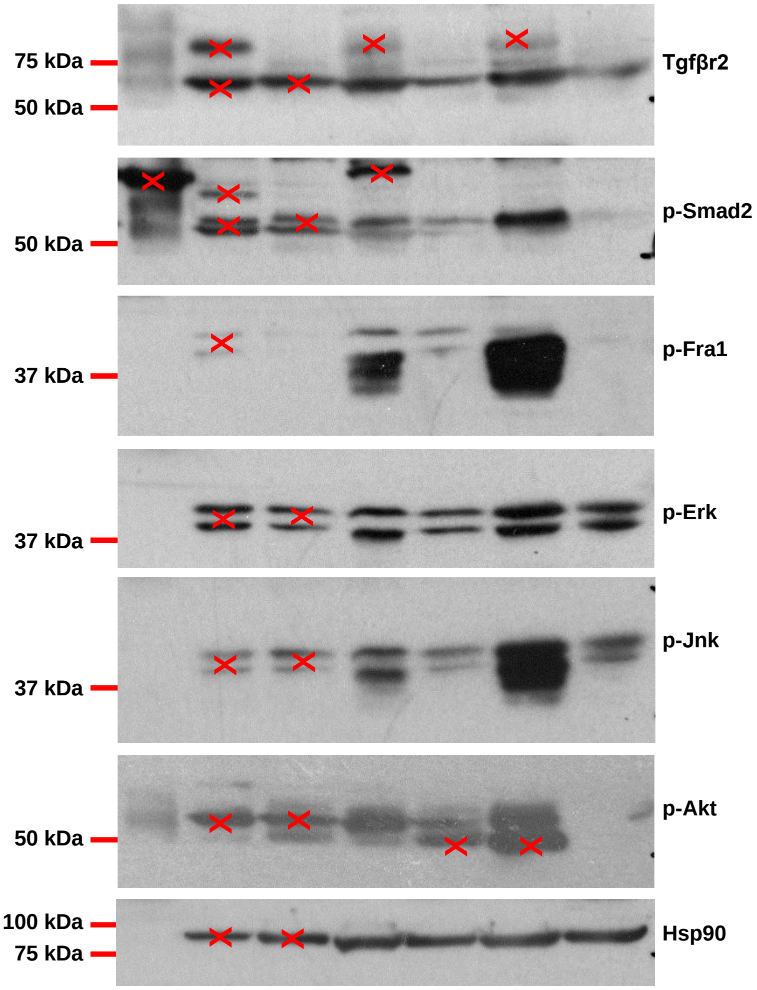

For Fig. 3A

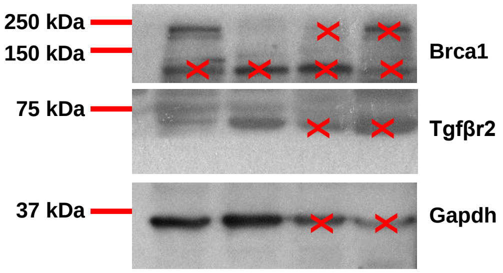

For Fig. 4A

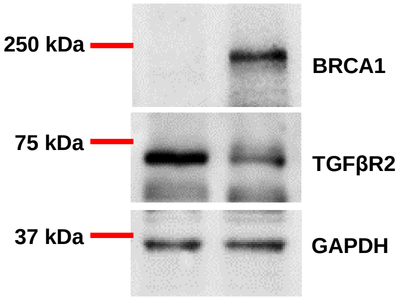

For Fig. 5A

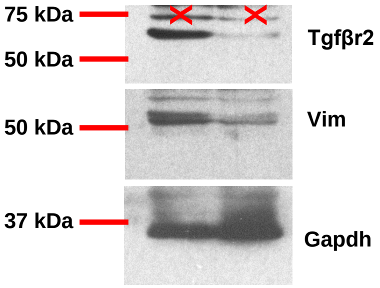

For Fig. 6A

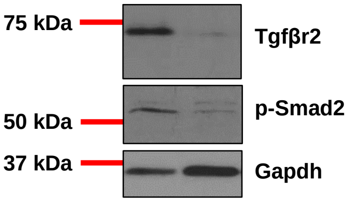

Fig. 9. Raw data for the blots included in each figure and supplementary data.

For Fig. 6D (left)

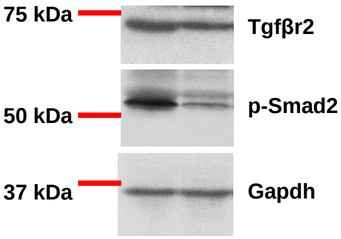

For Fig. 6D (right)

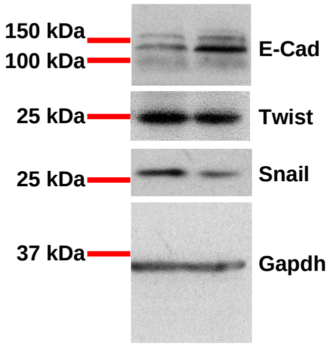

For Fig. S1

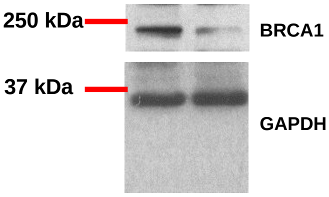

For Fig. S5

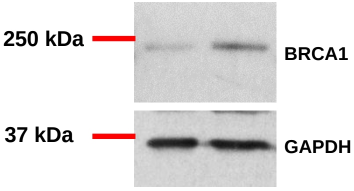

Supplement: Supplementary file 1 — Table S1 and Figure S1-S9 [file 41419_2022_4646_MOESM1_ESM.pdf]
