# Supplementary material for: Loss of function of BRCA1 promotes EMT in mammary tumors through activation of TGFβR2 signaling pathway
Source: Cell Death Dis. 2022 Mar 2;13(3):195. doi: 10.1038/s41419-022-04646-7 (PMC8891277; doi:10.1038/s41419-022-04646-7)
Supplement: Supplementary file 2 — Agreement emails to authorship change [file 41419_2022_4646_MOESM2_ESM.pdf]

深圳大学 邮件系统 SHENZHEN UNIVERSITY
锁屏 退出 邮件全文搜索
写信
收件箱
待办邮件
草稿箱 84
已发送
其他文件夹 112
收件箱 CDDIS-21-... RE: Final D... Re: Please ...
回复 回复全部 转发 移动到 标记为 更多 删除

Re: Please respond ASAP

Baifeng 发送给 裴新海

发起会议

2022-02-07 20:34:37

I agree to the authorship
Thanks
Feng Bai

发自我的 iPhone

在 2022年2月7日，下午8:27，<peixinhai@szu.edu.cn> <peixinhai@szu.edu.cn> 写道：

Dear co-authors:

Our below manuscript has been provisionally accepted for publication in *Cell Death & Disease*.

Manuscript Number: CDDIS-21-3554RR
Title: Loss of function of BRCA1 promotes EMT in mammary tumors through activation of TGFβR2 signaling pathway
Current authors are: Feng Bai, Chuying Wang, Xiong Liu, Daniel Hollern, Shiqin Liu, Cheng Fan, Chang Liu, Sijia Ren, Jason Herschkowitz, Wei-Guo Zhu, and Xin-Hai Pei

During pre-submission stage, the authors of the manuscript were: Feng Bai, Chuying Wang, Xiong Liu, Daniel Hollern, Shiqin Liu, Cheng Fan, Chang Liu, Sijia Ren, Jason Herschkowitz, Charles M. Perou, Wei-Guo Zhu, and Xin-Hai Pei.

Dr. Chuck Perou requested to remove his name from the manuscript, while the manuscript was in format “Initial Quality Check” stage in last Sep. Although Dr. Charles Perou’s name was removed from the author list per his own request before the manuscript was officially sent out for review, *Cell Death & Disease* still requests that every co-author confirm your agreement with the current authorship before the manuscript is officially accepted. Please reply to this email confirming that you agree to the current authorship.

Thank you.

快捷回复给所有人

深圳大学 邮件系统 SHENZHEN UNIVERSITY

锁屏 退出 邮件全文搜索

写信

收件箱
待办邮件
草稿箱 84
已发送
其他文件夹 112

收件箱 Re: Please ... ×

回复 回复全部 转发 移动到 标记为 更多 删除

Re: Please respond ASAP

发起会议
2022-02-08 12:21:14

发件人："王楚莹" <wangchuying0409@163.com>
收件人："裴新海" <peixinhai@szu.edu.cn>

I agree.

Chuying Wang

王楚莹
邮箱：wangchuying0409@163.com

On 02/07/2022 20:27, peixinhai wrote:

Dear co-authors:

Our below manuscript has been provisionally accepted for publication in *Cell Death & Disease*.

Manuscript Number: CDDIS-21-3554RR
Title: Loss of function of BRCA1 promotes EMT in mammary tumors through activation of TGFβR2 signaling pathway
Current authors are: Feng Bai, Chuying Wang, Xiong Liu, Daniel Hollern, Shiqin Liu, Cheng Fan, Chang Liu, Sijia Ren, Jason Herschkowitz, Wei-Guo Zhu, and Xin-Hai Pei

During pre-submission stage, the authors of the manuscript were: Feng Bai, Chuying Wang, Xiong Liu, Daniel Hollern, Shiqin Liu, Cheng Fan, Chang Liu, Sijia Ren, Jason Herschkowitz, Charles M. Perou, Wei-Guo Zhu, and Xin-Hai Pei.

Dr. Chuck Perou requested to remove his name from the manuscript, while the manuscript was in format "Initial Quality Check" stage in last Sep. Although Dr. Charles Perou's name was removed from the author list per his own request before the manuscript was officially sent out for review, *Cell Death & Disease* still requests that every co-author confirm your agreement with the current authorship before the manuscript is officially accepted. Please reply to this email confirming that you agree to the current authorship.

Thank you.

Xin-Hai

快捷回复给所有人

深圳大学 邮件系统 SHENZHEN UNIVERSITY
锁屏 退出 邮件全文搜索
写信
收件箱
待办邮件
草稿箱 84
已发送
其他文件夹 111
收件箱 CDDIS-21-... RE: Final D... FW: Final ... Re: Please ...
回复 回复全部 转发 移动到 标记为 更多 删除
发起会议

Re: Please respond ASAP

xiong_liu@szu.edu.cn 发送给 裴新海

2022-02-08 09:04:20

I agree to the authorship

Congratulations.

Thanks so much!

Xiong Liu

Best Wishes

发自我的iPhone

------------------ Original ------------------

From: peixinhai@szu.edu.cn <peixinhai@szu.edu.cn>
Date: Mon,Feb 7,2022 8:27 PM
To: baifeng <baifeng@szu.edu.cn>, '王楚莹' <wangchuying0409@163.com>, '刘雄' <xiong_liu@szu.edu.cn>, 'Hollern, Daniel' <hollernd@email.unc.edu>, 'Daniel Hollern' <dhollern@salk.edu>, 'Shiqin Liu' <shiqliu@stanford.edu>, 'Cheng Fan' <cfan2012@gmail.com>, '刘畅' <liuchang6@szu.edu.cn>, '任思嘉' <rensijia790651896@163.com>, 'Herschkowitz, Jason I' <jherschkowitz@albany.edu>, 'Dr. Wei-Guo Zhu' <zhuweiguo@szu.edu.cn>
Cc: peixinhai <peixinhai@szu.edu.cn>
Subject: Re: Please respond ASAP

Dear co-authors:

Our below manuscript has been provisionally accepted for publication in *Cell Death & Disease*.

Manuscript Number: CDDIS-21-3554RR
Title: Loss of function of BRCA1 promotes EMT in mammary tumors through activation of TGFβR2 signaling pathway
Current authors are: Feng Bai, Chuying Wang, Xiong Liu, Daniel Hollern, Shiqin Liu, Cheng Fan, Chang Liu, Sijia Ren, Jason Herschkowitz, Wei-Guo Zhu, and Xin-Hai Pei

During pre-submission stage, the authors of the manuscript were: Feng Bai, Chuying Wang, Xiong Liu, Daniel Hollern, Shiqin Liu, Cheng Fan, Chang Liu, Sijia Ren, Jason Herschkowitz, Charles M. Perou, Wei-Guo Zhu, and Xin-Hai Pei.

Dr. Chuck Perou requested to remove his name from the manuscript, while the manuscript was in format “Initial Quality Check” stage in last Sep. Although Dr. Charles Perou’s name was removed from the author list per his own request before the manuscript was officially sent out for review, *Cell Death & Disease* still requests that every co-author confirm your agreement with the current authorship before the manuscript is officially accepted. Please reply to this email confirming that you agree to the current authorship.

Thank you.

快捷回复给所有人

深圳大学 邮件系统 锁屏 退出 邮件全文搜索

写信
收件箱
待办邮件
草稿箱 84
已发送
其他文件夹 111

收件箱 | CDDIS-21-... | RE: Final D... | FW: Final ... | Re: Please ... | Re: Please ...

回复 回复全部 转发 移动到 标记为 更多 删除

Re: Please respond ASAP

Daniel Hollern 发送给 裴新海 , baifeng@szu.edu.cn , '王楚莹' , '刘雄' , 'Hollern, ...

发起会议
2022-02-08 01:25:31

I agree with the authorship

Daniel Hollern, PhD

ASSISTANT PROFESSOR, SALK CANCER CENTER

NOMIS CENTER FOR IMMUNOBIOLOGY AND MICROBIAL PATHOGENESIS

salk®

Salk Institute for Biological Studies

10010 N Torrey Pines Rd • La Jolla, CA 92037

M (616) 443-1548 • **E** dhollern@salk.edu

WWW.SALK.EDU

TWITTER • FACEBOOK • YOUTUBE • LINKEDIN • INSTAGRAM

Note: This email message (including any attachments) is for the sole use of the intended recipient(s) and may contain confidential, proprietary and privileged information. Any unauthorized review, use, disclosure or distribution is prohibited. If you are not the intended recipient, please contact the sender by reply email and destroy the original message along with any and all copies.

--

From: "peixinhai@szu.edu.cn" <peixinhai@szu.edu.cn>
Date: Monday, February 7, 2022 at 4:27 AM
To: "baifeng@szu.edu.cn" <baifeng@szu.edu.cn>, '王楚莹' <wangchuying0409@163.com>, '刘雄' <xiong_liu@szu.edu.cn>, "'Hollern, Daniel'" <hollernd@email.unc.edu>, Daniel Hollern <dhollern@salk.edu>, 'Shiqin Liu' <shiqliu@stanford.edu>, 'Cheng Fan' <cfan2012@gmail.com>, '刘畅' <liuchang6@szu.edu.cn>, '任思嘉' <rensijia790651896@163.com>, "'Herschkowitz, Jason I'" <jherschkowitz@albany.edu>, "'Dr. Wei-Guo Zhu'" <zhuweiguo@szu.edu.cn>
Cc: "peixinhai@szu.edu.cn" <peixinhai@szu.edu.cn>
Subject: Please respond ASAP

Dear co-authors:

Our below manuscript has been provisionally accepted for publication in *Cell Death & Disease*.

Manuscript Number: CDDIS-21-3554RR
Title: Loss of function of BRCA1 promotes EMT in mammary tumors through activation of TGFβR2 signaling pathway
Current authors are: Feng Bai, Chuying Wang, Xiong Liu, Daniel Hollern, Shiqin Liu, Cheng Fan, Chang Liu, Sijia Ren, Jason Herschkowitz, Wei-Guo Zhu, and Xin-Hai Pei

During pre-submission stage, the authors of the manuscript were: Feng Bai, Chuying Wang, Xiong Liu, Daniel Hollern, Shiqin Liu, Cheng Fan, Chang Liu, Sijia Ren, Jason Herschkowitz, Charles M. Perou, Wei-Guo Zhu, and Xin-Hai Pei.

Dr. Chuck Perou requested to remove his name from the manuscript, while the manuscript was in format “Initial Quality Check” stage in last Sep. Although Dr. Charles Perou’s name was removed from the author list per his own request before the manuscript was officially sent out for review, *Cell Death & Disease* still requests that every co-author confirm your agreement with the current authorship before the manuscript is officially accepted. Please reply to this email confirming that you agree to the current authorship.

Thank you.

快捷回复给所有人

深圳大学 邮件系统 SHENZHEN UNIVERSITY

锁屏 退出 邮件全文搜索

写信

收件箱
待办邮件
草稿箱 84
已发送
其他文件夹 112

收件箱 | CDDIS-21-... | RE: Final D... | Re: Please ...

回复 | 回复全部 | 转发 | 移动到 | 标记为 | 更多 | 删除

Re: Please respond ASAP

Shiqin Liu 发送给 裴新海

发起会议
2022-02-07 22:26:07

I agree.
Thank you,

Shiqin

From: Baifeng <baifeng@szu.edu.cn>
Sent: Monday, February 7, 2022 4:34:37 AM
To: peixinhai@szu.edu.cn <peixinhai@szu.edu.cn>
Cc: 王楚莹 <wangchuying0409@163.com>; 刘雄 <xiong_liu@szu.edu.cn>; Hollern, Daniel <hollernd@email.unc.edu>; Daniel Hollern <dhollern@salk.edu>; Shiqin Liu <shiqliu@stanford.edu>; Cheng Fan <cfan2012@gmail.com>; 刘畅 <liuchang6@szu.edu.cn>; 任思嘉 <rensijia790651896@163.com>; Herschkowitz, Jason I <jherschkowitz@albany.edu>; Dr. Wei-Guo Zhu <zhuweiguo@szu.edu.cn>
Subject: Re: Please respond ASAP

I agree to the authorship
Thanks
Feng Bai

发自我的 iPhone

在 2022年2月7日，下午8:27，<peixinhai@szu.edu.cn> <peixinhai@szu.edu.cn> 写道：

Dear co-authors:
Our below manuscript has been provisionally accepted for publication in *Cell Death & Disease*.

Manuscript Number: CDDIS-21-3554RR
Title: Loss of function of BRCA1 promotes EMT in mammary tumors through activation of TGFβR2 signaling pathway
Current authors are: Feng Bai, Chuying Wang, Xiong Liu, Daniel Hollern, Shiqin Liu, Cheng Fan, Chang Liu, Sijia Ren, Jason Herschkowitz, Wei-Guo Zhu, and Xin-Hai Pei

During pre-submission stage, the authors of the manuscript were: Feng Bai, Chuying Wang, Xiong Liu, Daniel Hollern, Shiqin Liu, Cheng Fan, Chang Liu, Sijia Ren, Jason Herschkowitz, Charles M. Perou, Wei-Guo Zhu, and Xin-Hai Pei.

Dr. Chuck Perou requested to remove his name from the manuscript, while the manuscript was in format “Initial Quality Check” stage in last Sep. Although Dr. Charles Perou’s name was removed from the author list per his own request before the manuscript was officially sent out for review. *Cell Death & Disease* still requests that every co-author confirm your agreement with the

快捷回复给所有人

深圳大学 邮件系统 SHENZHEN UNIVERSITY
锁屏 退出 邮件全文搜索
写信
收件箱
待办邮件
草稿箱 84
已发送
其他文件夹 112
收件箱 CDDIS-21-... RE: Final D... Re: Please ...
回复 回复全部 转发 移动到 标记为 更多 删除

Re: Please respond ASAP

Cheng Fan 发送给 裴新海

发起会议
2022-02-07 23:00:54

```
I agree with the authorship.

Thank you,
Cheng

On Mon, Feb 7, 2022 at 7:29 AM <peixinhai@szu.edu.cn> wrote:
```

Dear co-authors:

Our below manuscript has been provisionally accepted for publication in *Cell Death & Disease*.

Manuscript Number: CDDIS-21-3554RR
Title: Loss of function of BRCA1 promotes EMT in mammary tumors through activation of TGFβR2 signaling pathway
Current authors are: Feng Bai, Chuying Wang, Xiong Liu, Daniel Hollern, Shiqin Liu, Cheng Fan, Chang Liu, Sijia Ren, Jason Herschkowitz, Wei-Guo Zhu, and Xin-Hai Pei

During pre-submission stage, the authors of the manuscript were: Feng Bai, Chuying Wang, Xiong Liu, Daniel Hollern, Shiqin Liu, Cheng Fan, Chang Liu, Sijia Ren, Jason Herschkowitz, Charles M. Perou, Wei-Guo Zhu, and Xin-Hai Pei.

Dr. Chuck Perou requested to remove his name from the manuscript, while the manuscript was in format "Initial Quality Check" stage in last Sep. Although Dr. Charles Perou's name was removed from the author list per his own request before the manuscript was officially sent out for review, *Cell Death & Disease* still requests that every co-author confirm your agreement with the current authorship before the manuscript is officially accepted. Please reply to this email confirming that you agree to the current authorship.

Thank you.

[illegible]

快捷回复给所有人

深圳大学 邮件系统

锁屏 退出 邮件全文搜索

写信

收件箱
待办邮件
草稿箱 84
已发送
其他文件夹 112

收件箱 | CDDIS-21-... | RE: Final D... | Re:Fw:Plea...

回复 回复全部 转发 移动到 标记为 更多 删除

Re:Fw:Please respond ASAP

LC 发送给 裴新海

发起会议
2022-02-07 22:35:25

Dear Prof. Pei,

I agree with the current authorship.
Best,

Chang Liu

At 2022-02-07 21:52:15, "裴新海" <peixinhai@szu.edu.cn> wrote:

Hi, Dr. Chang Liu:
Please find the email re-sent to your new email address.
Please respond ASAP.
Xin-Hai

-------- 转发邮件信息 --------
发件人：peixinhai@szu.edu.cn
发送日期：2022-02-07 20:27:42
收件人：baifeng@szu.edu.cn, "'王楚莹'" <wangchuying0409@163.com>, "'刘雄'" <xiong_liu@szu.edu.cn>, "'Hollern, Daniel'" <hollernd@email.unc.edu>, "'Daniel Hollern'" <dhollern@salk.edu>, "'Shiqin Liu'" <shiqliu@stanford.edu>, "'Cheng Fan'" <cfan2012@gmail.com>, "'刘畅'" <liuchang6@szu.edu.cn>, "'任思嘉'" <rensijia790651896@163>
抄送人：peixinhai@szu.edu.cn
主 题：Please respond ASAP

Dear co-authors:

Our below manuscript has been provisionally accepted for publication in *Cell Death & Disease*.

Manuscript Number: CDDIS-21-3554RR
Title: Loss of function of BRCA1 promotes EMT in mammary tumors through activation of TGFβR2 signaling pathway
Current authors are: Feng Bai, Chuying Wang, Xiong Liu, Daniel Hollern, Shiqin Liu, Cheng Fan, Chang Liu, Sijia Ren, Jason Herschkowitz, Wei-Guo Zhu, and Xin-Hai Pei

During pre-submission stage, the authors of the manuscript were: Feng Bai, Chuying Wang, Xiong Liu, Daniel Hollern, Shiqin Liu, Cheng Fan, Chang Liu, Sijia Ren, Jason Herschkowitz, Charles M. Perou, Wei-Guo Zhu, and Xin-Hai Pei.

Dr. Chuck Perou requested to remove his name from the manuscript, while the manuscript was in format “Initial Quality Check” stage in last Sep. Although Dr. Charles Perou’s name was removed from the author list per his own request before the manuscript was officially sent out for review, *Cell Death & Disease* still requests that every co-author confirm your agreement with the current authorship before the manuscript is officially accepted. Please reply to this email confirming that you agree to the current authorship.

Thank you.

Xin-Hai

快捷回复给所有人

深圳大学 邮件系统 锁屏 退出 邮件全文搜索

写信
收件箱
待办邮件
草稿箱 84
已发送
其他文件夹 112

收件箱 | CDDIS-21-... | RE: Final D... | Re: Please ...

回复 | 回复全部 | 转发 | 移动到 | 标记为 | 更多 | 删除

Re: Please respond ASAP

rensijia790651896@163.com 发送给 裴新海

发起会议
2022-02-07 21:12:44

I agree to the authorship.
Thanks .
Sijia

---Original---

From: "peixinhai"<peixinhai@szu.edu.cn>
Date: Mon, Feb 7, 2022 13:27 PM
To: "baifeng"<baifeng@szu.edu.cn>;"'王楚莹'"<wangchuying0409@163.com>;"'刘雄'"<xiong_liu@szu.edu.cn>;"'Hollern, Daniel'"<hollernd@email.unc.edu>;"'Daniel Hollern'"<dhollern@salk.edu>;"'Shiqin Liu'"<shiqliu@stanford.edu>;"'Cheng Fan'"<cfan2012@gmail.com>;"'刘畅'"<liuchang6@szu.edu.cn>;"'任思嘉'"<rensijia790651896@163.com>;"'Herschkowitz, Jason I'"<jherschkowitz@albany.edu>;"'Dr. Wei-Guo Zhu'"<zhuweiguo@szu.edu.cn>;
Cc: "peixinhai"<peixinhai@szu.edu.cn>;
Subject: Please respond ASAP

Dear co-authors:

Our below manuscript has been provisionally accepted for publication in *Cell Death & Disease*.

Manuscript Number: CDDIS-21-3554RR
Title: Loss of function of BRCA1 promotes EMT in mammary tumors through activation of TGFβR2 signaling pathway
Current authors are: Feng Bai, Chuying Wang, Xiong Liu, Daniel Hollern, Shiqin Liu, Cheng Fan, Chang Liu, Sijia Ren, Jason Herschkowitz, Wei-Guo Zhu, and Xin-Hai Pei

During pre-submission stage, the authors of the manuscript were: Feng Bai, Chuying Wang, Xiong Liu, Daniel Hollern, Shiqin Liu, Cheng Fan, Chang Liu, Sijia Ren, Jason Herschkowitz, Charles M. Perou, Wei-Guo Zhu, and Xin-Hai Pei.

Dr. Chuck Perou requested to remove his name from the manuscript, while the manuscript was in format "Initial Quality Check" stage in last Sep. Although Dr. Charles Perou's name was removed from the author list per his own request before the manuscript was officially sent out for review, *Cell Death & Disease* still requests that every co-author confirm your agreement with the current authorship before the manuscript is officially accepted. Please reply to this email confirming that you agree to the current authorship.

Thank you.

快捷回复给所有人

深圳大学 邮件系统
锁屏 退出 邮件全文搜索
写信
收件箱
待办邮件
草稿箱 84
已发送
其他文件夹 112
收件箱 CDDIS-21-... RE: Final D... RE: Please ...
回复 回复全部 转发 移动到 标记为 更多 删除
发起会议

RE: Please respond ASAP

Herschkowitz, Jason I 发送给 裴新海 , baifeng@szu.edu.cn , '王楚莹' ,...

2022-02-08 00:41:40

I agree with the current authorship.

-Jason Herschkowitz

From: peixinhai@szu.edu.cn <peixinhai@szu.edu.cn>
Sent: Monday, February 7, 2022 7:28 AM
To: baifeng@szu.edu.cn; '王楚莹' <wangchuying0409@163.com>; '刘雄' <xiong_liu@szu.edu.cn>; 'Hollern, Daniel' <hollernd@email.unc.edu>; 'Daniel Hollern' <dhollern@salk.edu>; 'Shiqin Liu' <shiqliu@stanford.edu>; 'Cheng Fan' <cfan2012@gmail.com>; '刘畅' <liuchang6@szu.edu.cn>; '任思嘉' <rensijia790651896@163.com>; Herschkowitz, Jason I <jherschkowitz@albany.edu>; 'Dr. Wei-Guo Zhu' <zhuweiguo@szu.edu.cn>
Cc: peixinhai@szu.edu.cn
Subject: Please respond ASAP

Dear co-authors:

Our below manuscript has been provisionally accepted for publication in *Cell Death & Disease*.

Manuscript Number: CDDIS-21-3554RR
Title: Loss of function of BRCA1 promotes EMT in mammary tumors through activation of TGFβR2 signaling pathway
Current authors are: Feng Bai, Chuying Wang, Xiong Liu, Daniel Hollern, Shiqin Liu, Cheng Fan, Chang Liu, Sijia Ren, Jason Herschkowitz, Wei-Guo Zhu, and Xin-Hai Pei

During pre-submission stage, the authors of the manuscript were: Feng Bai, Chuying Wang, Xiong Liu, Daniel Hollern, Shiqin Liu, Cheng Fan, Chang Liu, Sijia Ren, Jason Herschkowitz, Charles M. Perou, Wei-Guo Zhu, and Xin-Hai Pei.

Dr. Chuck Perou requested to remove his name from the manuscript, while the manuscript was in format "Initial Quality Check" stage in last Sep. Although Dr. Charles Perou's name was removed from the author list per his own request before the manuscript was officially sent out for review, *Cell Death & Disease* still requests that every co-author confirm your agreement with the current authorship before the manuscript is officially accepted. Please reply to this email confirming that you agree to the current authorship.

Thank you.

快捷回复给所有人

深圳大学 邮件系统
锁屏 退出 邮件全文搜索
写信
收件箱
待办邮件
草稿箱 84
已发送
其他文件夹 111
收件箱 CDDIS-21-... RE: Final D... FW: Final ...
回复 回复全部 转发 移动到 标记为 更多 删除

FW: Final Decision made for CDDIS-21-3554

Perou, Charles M 发送给 裴新海

2021-09-20 04:47:45

发起会议

Xin-Hai,

This paper is getting the total run around and now I see the editors are asking for 100 new things even before they send it out for review. I must say this paper has so many new things in it, all of which I have had no part of. I no longer feel I should be an author on this as my contribution is now so slight, and it has evolved so much from when I last actually did anything that I can barely recognize it relative to where it started. I request that next time it is submitted, that I be removed as an author, but you are of course free to continue to use and publish on the small amount of genomic data that is unique to this paper. I wish you the best of luck with this paper, CHUCK

From: cddisease@springernature.com <cddisease@springernature.com>
Sent: Tuesday, September 14, 2021 3:52 AM
To: Perou, Charles M <chuck_perou@med.unc.edu>
Subject: Final Decision made for CDDIS-21-3554

Dear Dr. Perou:

Here is a copy of the decision letter for manuscript "Loss of function of BRCA1 promotes EMT in mammary tumors through activation of TGFβR2 signaling pathway" by Feng Bai, Chuying Wang, Xiong Liu, Daniel Hollern, Shiqin Liu, Cheng Fan, Chang Liu, Sijia Ren, Jason Herschkowitz, Charles Perou, Wei-Guo Zhu, and Xin-Hai Pei [Paper #CDDIS-21-3554], which you were a Contributing Author.

You can now use a single sign-on for all your accounts, view the status of all your manuscript submissions and reviews, access usage statistics for your published articles and download a record of your refereeing activity for the Nature journals.

In addition, NPG encourages all authors and reviewers to associate an Open Researcher and Contributor Identifier (ORCID) to their account. ORCID is a community-based initiative that provides an open, non-proprietary and transparent registry of unique identifiers to help disambiguate research contributions.

Sincerely,

Mauro Piacentini, Yufang Shi and Hans-Uwe Simon
Editors-in-Chief,
Cell Death & Disease

Subject: CDDIS-21-3554decision letter

Dear Professor Pei
RE: Manuscript CDDIS-21-3554, Loss of function of BRCA1 promotes EMT in mammary tumors through activation of TGFβR2 signaling pathway by Professor Pei

Many thanks for sending us the above-mentioned manuscript which has now been seen by the receiving editor.

CDDis is willing to send the paper you describe for in-depth review but as is always true, we cannot guarantee that the outcome of the peer review process will be positive and that we will publish the paper. To enable our reviewers to assess your manuscript effectively and efficiently, the authors should revise manuscript.

Major comments
1) Number of replicates/samples for statistical analyses
The authors also should note the number of samples/replicates (n=..) in the Figure legends.

2) Student t-test
The authors should note whether paired or unpaired student t-test were utilized for these statistical analyses.

3) Whole blots of western blotting
There are a lot of western blotting images shown in this manuscript. The authors should present WHOLE untrimmed western blotting images as supplementary data. If the authors trimmed blots during experiments, the authors should repeat the experiments. It should be noted that CDDis will NOT compromise the image

快捷回复给所有人

主　题:	Re: RE: Re: Please respond ASAP
发件人:	"朱卫国" <zhuweiguo@szu.edu.cn> 2022-2-8 11:29:56
收件人:	peixinhai@szu.edu.cn

I agree!

-----原始邮件-----
发件人:peixinhai@szu.edu.cn
发送时间:2022-02-08 09:06:37 (星期二)
收件人: '"朱卫国"' <zhuweiguo@szu.edu.cn>
抄送:
主题: RE: Re: Please respond ASAP

Thanks.

Xin-Hai

From: 朱卫国 <zhuweiguo@szu.edu.cn>
Sent: 2022年2月7日 22:48
To: Shiqin Liu <shiqliu@stanford.edu>
Cc: peixinhai@szu.edu.cn; 王楚莹 <wangchuying0409@163.com>; 刘雄 <xiong_liu@szu.edu.cn>; Hollern, Daniel <hollernd@email.unc.edu>; Daniel Hollern <dhollern@salk.edu>; Cheng Fan <cfan2012@gmail.com>; 刘畅 <liuchang6@szu.edu.cn>; 任思嘉 <rensijia790651896@163.com>; Herschkowitz, Jason I <jherschkowitz@albany.edu>; Baifeng <baifeng@szu.edu.cn>
Subject: Re: Re: Please respond ASAP

I agree with this request. Thank you.

Best,

Zhu WG

-----原始邮件-----
发件人:"Shiqin Liu" <shiqliu@stanford.edu>
发送时间:2022-02-07 22:26:07 (星期一)
收件人: "peixinhai@szu.edu.cn" <peixinhai@szu.edu.cn>
抄送: "王楚莹" <wangchuying0409@163.com>, "刘雄" <xiong_liu@szu.edu.cn>, "Hollern, Daniel" <hollernd@email.unc.edu>, "Daniel Hollern" <dhollern@salk.edu>, "Cheng Fan" <cfan2012@gmail.com>, "刘畅" <liuchang6@szu.edu.cn>, "任思嘉" <rensijia790651896@163.com>, "Herschkowitz, Jason I" <jherschkowitz@albany.edu>, "Dr. Wei-Guo Zhu" <zhuweiguo@szu.edu.cn>, Baifeng <baifeng@szu.edu.cn>
主题: Re: Please respond ASAP

I agree.

Thank you,

Shiqin

From: Baifeng <baifeng@szu.edu.cn>
Sent: Monday, February 7, 2022 4:34:37 AM
To: peixinhai@szu.edu.cn <peixinhai@szu.edu.cn>
Cc: 王楚莹 <wangchuying0409@163.com>; 刘雄 <xiong_liu@szu.edu.cn>; Hollern, Daniel <hollernd@email.unc.edu>; Daniel Hollern <dhollern@salk.edu>; Shiqin Liu <shiqliu@stanford.edu>; Cheng Fan <cfan2012@gmail.com>; 刘畅 <liuchang6@szu.edu.cn>; 任思嘉 <rensijia790651896@163.com>; Herschkowitz, Jason I <jherschkowitz@albany.edu>; Dr. Wei-Guo Zhu <zhuweiguo@szu.edu.cn>
Subject: Re: Please respond ASAP

I agree to the authorship

Thanks

Feng Bai

发自我的 iPhone

在 2022年2月7日，下午8:27，<peixinhai@szu.edu.cn> <peixinhai@szu.edu.cn> 写道：

Dear co-authors:

Our below manuscript has been provisionally accepted for publication in *Cell Death & Disease*.

Manuscript Number: CDDIS-21-3554RR
Title: Loss of function of BRCA1 promotes EMT in mammary tumors through activation of TGFβR2 signaling pathway
Current authors are: Feng Bai, Chuying Wang, Xiong Liu, Daniel Hollern, Shiqin Liu, Cheng Fan, Chang Liu, Sijia Ren, Jason Herschkowitz, Wei-Guo Zhu, and Xin-Hai Pei

During pre-submission stage, the authors of the manuscript were: Feng Bai, Chuying Wang, Xiong Liu, Daniel Hollern, Shiqin Liu, Cheng Fan, Chang Liu, Sijia Ren, Jason Herschkowitz, Charles M. Perou, Wei-Guo Zhu, and Xin-Hai Pei.

Dr. Chuck Perou requested to remove his name from the manuscript, while the manuscript was in format "Initial Quality Check" stage in last Sep. Although Dr. Charles Perou's name was removed from the author list per his own request before the manuscript was officially sent out for review, *Cell Death & Disease* still requests that every co-author confirm your agreement with the current authorship before the manuscript is officially accepted. Please reply to this email confirming that you agree to the current authorship.

Thank you.

Xin-Hai
